# Bacterial Communities Are More Sensitive to Water Addition Than Fungal Communities Due to Higher Soil K and Na in a Degraded Karst Ecosystem of Southwestern China

**DOI:** 10.3389/fmicb.2020.562546

**Published:** 2020-11-09

**Authors:** Muhammad Umair, Ningxiao Sun, Hongmei Du, Nan Hui, Muhammad Altaf, Baoming Du, Shan Yin, Chunjiang Liu

**Affiliations:** ^1^School of Agriculture and Biology, Shanghai Jiao Tong University, Shanghai, China; ^2^Shanghai Urban Forest Research Station, State Forestry and Grassland Administration, Shanghai, China; ^3^Key Laboratory of Urban Agriculture (South), Ministry of Agriculture, Shanghai, China; ^4^School of Design, Shanghai Jiao Tong University, Shanghai, China; ^5^Department of Zoology, Women University of Azad Jammu and Kashmir, Bagh, Pakistan

**Keywords:** climate change, karst regions, watering treatment, soil elements, soil microbial communities

## Abstract

Precipitation is predicted to become more intense in Southern China in the context of climate change; however, the responses of microbial communities to variations in soil moisture have not been well documented for karst areas. The climate is typically in a subtropical monsoon category with two different seasons: a dry season (December–May) and a wet season (June–November). Based on a randomized complete block design (RCBD), a water addition experiment (0, +20, +40, and +60% relative to local precipitation) was established in April 2017, with five replicates, in a degraded grass-shrub community. Sampling was performed in May and at the end of August of 2017. Macroelements (C, H, N, P, K, Ca, Mg, and S), microelements (Mn, Fe, Zn, and Cu), and non-essential elements (Na, Al, and Si) were quantified in the soil. The total DNA of the soil samples was analyzed through 16S rRNA amplicon by Illumina Miseq. Subsequent to the addition of water during both the dry and wet seasons, the concentrations of non-metal elements (C, H, N, S, and P, except for Si) in the soil remained relatively stable; however, metal elements (K, Na, Fe, and Mg, along with Si) increased significantly, whereas Zn and Ca decreased. During the dry season, fungal and bacterial communities were significantly distinct from those during the wet season along the PC axis 1 (*p* < 0.001). Water addition did not alter the compositions of bacterial or fungal communities during the dry season. However, during the wet season, water addition altered the compositions of bacterial rather than fungal community based on principal component analysis. At the phylum level, the relative abundance of Actinobacteria increased with water addition and had a significantly positive correlation with K^+^ (*r*^2^ = 0.70, *p* < 0.001) and Na^+^ (*r*^2^ = 0.36, *p* < 0.01) contents, whereas that of Acidobacteria, Planctomycetes, and Verrucomicrobia decreased and showed negative correlation with soil K and Na content, and no changes were observed for the fungal phyla. This suggests that the karst bacterial communities can be influenced by the addition of water during the wet season likely linked to changes in soil K and Na contents. These findings implied that increased rainfall might alter the elemental compositions of karst soils, and bacterial communities are likely to be more sensitive to variations in soil moisture in contrast to their fungal counterparts.

## Introduction

Global climate change may have considerable impacts on hydrological cycles worldwide, which may result in increased precipitation, higher evaporation rates, and the uneven distribution of rainfall (Nijssen et al., 2001; Lian et al., 2015). Some regions of the globe may witness significant changes in the timing of dry and wet seasons, which might trigger increases in both droughts and floods (Su et al., 2005; Zhang et al., 2013; Lian et al., 2015). Global warming poses serious threats to agricultural and natural systems, partly through its capacity to alter soil microbial communities and ecological processes due to variations in the content of soil moisture (Li et al., 2017). Specifically, climate changes influence the compositions of plant and soil communities; thus, they impact the functionality and services of natural ecosystems (Cregger et al., 2012; Anderson-Teixeira et al., 2013). Moreover, global warming poses a severe threat to karst hydrogeology and affects the magnitude of soil microbial biomass carbon in the karst areas of Southwestern China (Piao et al., 2000). In fact, immense diversity of microorganisms that live belowground contributes significantly to shaping aboveground biodiversity and the functioning of terrestrial ecosystems (Bardgett and Putten, 2014). However, it is essential to accurately elucidate the impacts of higher precipitation on microbial communities, as well as key ecological processes in soils.

In Southwestern China, the karst area of Yunnan Province covers 33.3 thousand km^2^, about 8.6% of the total area of the Province (Jiang et al., 2014). In Jianshui, a typical karst area located in Yunnan province, anthropogenic disturbances, e.g., forest clear cutting, grazing, and tilling, have pushed primary forests to the KRD shrubs during 1960s–1990s. A large portion of the degraded karst lands that exist in Southwestern China is the result of geo-ecological destruction in conjunction with significant anthropogenic disturbances such as agricultural expansion, urban sprawl, livestock grazing, and firewood production. Extensive land degradation and the deterioration of vegetation in these karst regions set the stage for extreme soil erosion, which create rocky landscapes. Fragile karst topographies/environments are formed through irrational and intensive land-use practices that result in their geo-ecological destruction (Wang et al., 2004; Yan and Cai, 2015). The fragility of these areas is based on the following facts: (i) Rocky karst substrates (e.g., carbonate rocks) are created in marine ecosystems, whose major elements are Mg, Ca, O, and C, while soils are comprised primarily of Al, Fe, and Si (Li and Cao, 2015). (ii) As Ca-rich carbonate rocks are soluble, their dissolution rates are one to two times higher than that of silicate mineral rocks, and the pH values of limestone soil range from 6.22 to 7.63 (Jianhua et al., 2015). (iii) Water shortages and the porosity of soils in karst ecosystems are not suitable for the survival of plants and soil organisms, which translates to the reduction in aboveground and belowground biomass (Daoxian, 2001; Jianhua et al., 2015; Li and Cao, 2015).

Most studies on karst rocky desertification have shown that land degradation reduced the plant diversity and soil quality (Qi et al., 2017; Dai et al., 2018). However, much less attention has been paid to the effects of climate change on soil microbes of karst areas, which are essential for the maintenance of vegetation and environmental restoration. Soil microbes are considered as one of the key regulators of soil ecosystems (Young and Crawford, 2004). They are the critical drivers of nutrient cycling in natural environments and rely on soil moisture to complete their activities and life spans (Vos et al., 2013; Meisner et al., 2018). Notably, soil microbial communities primarily account for nutrient mineralization and the decomposition of organic substances in ambient environments (Schimel, 1995; Cregger et al., 2012). Moreover, the compositions, structures, abundance, and activities of microbial communities are directly impacted by climatic factors (Williams and Rice, 2007; Stres et al., 2008; Cruz-Martínez et al., 2012). In fact, fluctuations in rainfall regimes can shift soil microbial communities by altering their structures and compositions through the regional loss of specific operational taxonomic units (OTUs; Fierer et al., 2003; Clark et al., 2009).

Seasonal changes in precipitation may have significant effects on the diversity, abundance, and composition of soil microbial communities in natural ecosystems (Schadt et al., 2003; Lipson and Schmidt, 2004; Hullar et al., 2006). Moreover, soil microbial communities structure and composition are more responsive to seasonal fluctuations in rainfall than precipitation treatments (Cregger et al., 2012). Seasonal rainfall cycles alter the moisture content of soils and, consequently, modify systemic water distribution, which may augment the impacts of altered precipitation on soil dwelling microbial communities (Hawkes et al., 2011; Yan et al., 2015). Multiple studies have reported that during the dry season, soil-residing microbial communities are active but gradually modify their compositions during the rainy season (Kavamura et al., 2013; Barnard et al., 2015; Taketani et al., 2017). Moreover, the responses of microbial communities to drying and rewetting periods vary with soil structures due to changes in moisture, chemical composition, and the bioavailability of organic matter in soils (Anderson and Ingram, 1994). However, the effects of drying and rewetting on soil microbial processes may directly influence nutrient cycling and the decomposition of soil organic matter (Fierer and Schimel, 2003; Schimel et al., 2007).

Soil pH, total organic carbon, and potassium and sodium concentrations are major factors correlating with soil microbial communities in karst ecosystems (Yun et al., 2016; Qi et al., 2018). Potassium and sodium, which can affect the transporter’s functions through controlling and cytoplasmic rehydration and ionic strength (Wood, 1999; Saxena et al., 2015), can also affect soil microbial community composition and diversity (Andronov et al., 2012; Pereira et al., 2014; Li et al., 2015). Such chemical components played a major role in determining the shape of the microbial community in natural ecosystems (Pereira et al., 2014). The addition of Na and K increased the mass production of the cellulase enzymes that are required to break down C-rich macromolecules by microorganisms (Kaspari et al., 2008; Kaspari et al., 2009). Moreover, the resilience, functions, and stability of ecosystems are determined by the interaction of soil microbial community and soil characteristics. However, these relations are largely unclear in karst ecosystems.

Bacteria and fungi are the most dominant in the soil inhabitants and also known to improve soil structure by promoting the formation of soil aggregates and nutrients bioavailability (Degens, 1997; Rashid et al., 2016). Specifically, soil moisture is a prominent indicator of fungal and bacterial community structures (Li et al., 2017), which directly influences microbial activities (Meisner et al., 2015). Changes in soil moisture due to fluctuations in rainfall alter the conditions for soil biota, which cause shifts in the structural diversity and functionality of soil-resident microbial communities (Fierer and Jackson, 2006; Kim et al., 2008). Based on multiple studies, the net annual precipitation in Southwestern China is anticipated to increase (Piao et al., 2010; Qin et al., 2015), which is likely to have significant impacts on the soil moisture of this region. Indeed, soil moisture is positively linked to the number of bacterial species in the soil, which is a good indicator of changes in the relative frequencies of species between soils (Dennis et al., 2019). Such information is particularly important for the regeneration of the degraded karst ecosystems, as the high bacterial diversity in the moist soil can significantly benefit the development and growth of tree seedlings (Santoyo et al., 2016). Research taking a more systematic approach to quantifying the impact of increasing precipitation on bacterial and fungal communities in degraded karst environments is also urgently required.

For this study, we conducted a controlled precipitation experiment in a degraded shrub- and grass-dominated community in a typical karst area of Southwestern China to mimic expected global warming-induced precipitation scenarios. The main aim of this study is to determine the effects of increased precipitation on soil characteristics and microbial communities in degraded karst ecosystems during both the dry and wet seasons. We hypothesized that (i) soil microbial community composition differs between watering treatments, which is directly affected by the variations in moisture content, and (ii) increased precipitation affects the interactions between soil microbial community composition and the soil characteristics.

## Materials and Methods

### Description of Study Area

This study was based on a field experiment that was conducted in a degraded shrub- and grass-dominated community at the Karst Ecosystem Research Station (KERS) of Jianshui City (23°59′N, 102°93′E) in Southwestern China. The climate of this area may be categorized as having two seasons: the wet season (June–November) with 85% annual precipitation and the dry season (December–May) with 15% annual precipitation. The annual average precipitation ranges 500–1,300 mm in Southwestern China during the time series from 1958 to 1988 (Prieler, 2005). During the period of 1981–2010, the average annual rainfall of this area is 770 mm^1^, with the highest annual precipitation of 1,096 mm in 1999. Annual precipitation showed slightly and statistically insignificant increasing trend, but statistically significant increasing trend has been detected in wet season (Qin et al., 2010). The median annual temperature is 20°C, with an average minimum of 14.6°C and average maximum of 24.6°C (Supplementary Figure 1), while the typical relative humidity is 71.8% (2010–2017). The average pH of the surface soil is 6.29 (1 M KCl; Umair et al., 2019, 2020).

### Experimental Design

A controlled precipitation experiment using a randomized complete block design (RCBD), which included five subblocks, was conducted in 20 sample plots (3 × 3 m in size) from the beginning of April in 2017 (Supplementary Figure 2). Each plot was separated from its neighboring plot by a distance of 3 m. Zhang et al. (2019), who designed the experiment for examining the impacts of increased precipitation on soil ecosystems, described the appropriate methods for measuring precipitation. Accordingly, we designed a four-level watering treatment regime CK (0%; control), T1 (+20%), T2 (+40%), and T3 (+60%), relative to the average monthly rainfall. The average monthly rainfall and temperature was measured on the basis of 2010–2017 climatic data, which was collected at a nearby meteorological station. The ambient rainfall was 143 mm for the dry season and 445 mm for the wet season in 2017. The daily variations in air temperature and precipitation were obtained by referencing nearby meteorological observation stations (Supplementary Figures 3, 4).

The watering treatments were set up through the addition of natural precipitation in April 2017, where the exact values of artificial rainfalls during the dry and wet seasons were 172 and 534 mm for T1, 200 and 623 mm for T2, and 228 and 712 mm for T3. The control (CK) plots received ambient rainfall only. The irrigation water was manually sprayed onto the water-treated plots three times each month, in the mornings of days 10, 20, and 30. The water was slowly and evenly sprayed over each plot such that run-off from the plot was avoided.

### Soil Sampling

Soil samples were collected after the water addition events during both the dry and wet seasons to observe seasonal trends in the compositions and diversity of soil microbial communities due to increased precipitation. Combined with the watering treatments, the wet season demonstrated evolved compositional changes in the microbial communities. It has been found that soil microbes are biologically more active in wet soil (Curiel Yuste et al., 2007). The soil was sampled using a soil corer (Ø2.5 cm × 0–10 cm deep, excluding the litter layer) in May (dry season) and August (wet season) of 2017, from near the trunks of trees/shrubs in each treatment plot. The soil samples for microbial analysis were loaded into polyethylene bags on dry ice in the field, until frozen at −20°C in the laboratory. Meanwhile, additional soil samples were collected and sealed in aluminum tins for chemical composition measurements. All of the soil samples were packed and transported to the experimental center of Shanghai Jiao Tong University, China. The soil was sieved to remove roots, stones, and large particles and stored in the laboratory at 25°C.

### Elemental Analysis

The concentrations of C, N, H, and S were determined from 25 mg of each sample using a powerful stable isotope ratio mass spectrometer (Vario EL III Element Analyzer; Elementar, Germany).

To quantify the concentrations of Fe, P, K, Mn, Mg, Ca, Na, Al, Cu, and Si, the samples were digested in a microwave reaction chamber under high temperature. The soil samples were placed in an oven at 105°C for 15 min and then dried at 60°C for 48 h. Then, all the samples were machine ground into powder. The soil samples passed through a 60-mesh sieve and were used for the determination of the total amount of soil elements. A 250-mg volume of sample powder was transferred to a 50-ml Teflon tube with 2 ml of HNO_3_ and 1 ml of H_2_O_2_. The mixture was heated at 105°C for 2 h with a Digiprep-MS digestion block (SCP Science, Champlain, NY, United States). The digested samples in 50-ml flasks were refilled with distilled water to a 40-ml volume. The elemental concentrations were quantified using an inductively coupled plasma-optical emission spectrometer (ICP-OES; Thermo Jarrell Ash IRIS Advantage 1000, Franklin, MA, United States) at the Shanghai Jiao Tong University Testing Center, China.

### DNA Extraction, Amplification, and Sequencing

The total DNA was extracted from approximately 0.5 *g* of soil using the Fast DNA SPIN extraction kit (MP Biomedicals, Santa Ana, CA, United States) according to the manufacturer’s protocol. The DNA yield was visualized via 1.0% agarose gel electrophoresis [1 × Tris–acetate–ethylenediaminetetraacetic acid (TAE) electrophoresis buffer run at 120 V for 1 h] with ethidium bromide (EtBr). The quality and quantity of extracted DNA was quantified using a Nano-Drop ND-1000 spectrophotometer (Thermo Fisher Scientific, Waltham, MA, United States). The extracted DNA was stored at −20°C prior to PCR amplification.

The PCR amplification of the V3–V4 region of the bacterial 16S rRNA genes was performed using the forward primer 338F (5′-ACTCCTACGGGAGGCAGCA-3′) and the reverse primer 806R (5′-GGACTACHVGGGTWTCTAAT-3′). For fungal internal transcribed spacer (ITS) amplification, a similar approach was employed using the forward primers fITS7 5′-GTGARTCATCGAATCTTTG-3′ and the reverse primer ITS4 5′-TCCTCCGCTTATTGATATGC-3′. Sample-specific 7-bp barcodes were incorporated into the primers for multiplex sequencing.

The PCR reactions were performed as described by Koskinen et al. (2011). The amplicons of the PCR were cleansed with Agencourt AMPure XP Beads (Beckman Coulter Inc., Indianapolis, IN, United States) and measured using the PicoGreen dsDNA Quantitation Assay (Molecular Probes-Invitrogen, Carlsbad, CA, United States). The amplicons of PCR were paired for equivalency following the quantification analysis of an individual. The libraries were sequenced using the Illumina MiSeq platform with MiSeq Reagent Kit v3 at the Shanghai Personal Biotechnology Co., Ltd (Shanghai, China), which produced pair-end 2 × 300-bp reads. The paired fastq files are available in the Sequence Read Archive at the National Center for Biotechnology Information^2^ under accession numbers SAMN14986759–SAMN14986778 for bacteria and SRR11826156–SRR11826157 for fungi, respectively.

#### Bioinformatics

Paired end sequence data (.fastq) were processed using mothur version 1.38.1 (Schloss et al., 2009). Both fungal and bacterial.fastq data files were contiged, and any sequences with ambiguous bases, more than one mismatch with the primers, homopolymers longer than 8 bp (bacteria) and 13 bp (fungi), or any without a minimum overlap of 50 bp were removed.

Bacterial sequences were aligned against a SILVA reference, preclustered to remove erroneous reads (Huse et al., 2010), screened for chimeras with the Vsearch algorithm (Rognes et al., 2016). Non-chimeric sequences were assigned to taxa using the Naive Bayesian Classifier (Wang et al., 2007) against the RDP training set (version 10). Non-target sequences (mitochondria, chloroplast, and Archaea) were removed. Sequences were clustered to OTUs at 97% similarity using the nearest neighbor (single-linkage) joining.

Fungal sequences were screened using the Vsearch algorithm, and putative chimeras were removed. To permit the pairwise alignment of fungal ITS sequences to calculate a pairwise distance matrix, we omitted all fungal ITS sequences that were <300 bp in length and truncated the remaining sequences to the first 300 bp. These fungal sequences were assigned to taxa using the Naive Bayesian Classifier and the UNITE-curated International Nucleotide Sequence Database reference database (Abarenkov et al., 2010). Any sequences not assigned to Kingdom Fungi were removed. Unique sequences were pairwise aligned and the resultant distance matrix clustered to OTUs at a 97% threshold using nearest neighbor joining as described for bacteria. Fungal OTUs were assigned to the functional guild using the FUNGuild database (Nguyen et al., 2016).

In both bacterial and fungal datasets, relatively low-abundance OTUs were deleted (≤10 sequences across all experimental units), as they may be sequencing or PCR artifacts (Tedersoo et al., 2010; Brown et al., 2015; Oliver et al., 2015). We calculated diversity and richness metrics for both bacterial and fungal communities in mothur. The observed OTU richness (S_obs_), the complement of Simpson’s diversity index (1/D: 1/Σp_*i*_^2^), Simpson’s evenness index (E_D_: 1/Σp_i_^2^/S), and Shannon’s diversity index (H′:Σp_i_lnp_i_), with p_i_ representing the frequency of each OTU within a sample, were iteratively calculated and rarefied at 19,322 sequences for bacteria and 18,981 for fungi.

### Soil pH and Soil Water Content Measurement

A digital pH meter was used to measure the soil pH in 1:5 soil/1 M KCl solutions (Mettler Toledo FE20/El20, Shanghai, China). The soil water content (SWC) was calculated by using the oven-drying method. Soil samples were oven dried for 48 h at 105°C. The SWC (%) was calculated by using the following formula:

$$\begin{array}{c} \text{SWC}\;\left(\%\right)=\frac{\text{W}1\;-\mathrm{W2}}{\text{W}2\;-\mathrm{W3}}\;\times 100 \end{array}$$

where W1 is the weight of wet soil plus box, W2 represents the weight of dry soil plus box, and W3 represents box weight.

HOBO® Micro Station Data Loggers (H21-002) were installed to check the soil temperature (°C) and volumetric soil moisture content (m^3^/m^3^) during the study period. Soil moisture conditions were monitored using an automatic soil moisture smart sensor (HOBO Micro Station #H21-002 Data Logger; Onset Computer Corp., Bourne, MA, United States; #S-SMx-M005; Supplementary Figure 5). To measure the soil temperature, HOBO 12-bit temperature smart sensors (S-TMB-M0xx) were connected to HOBO Micro Station data loggers. Automatic measurements were obtained every 10 s (Supplementary Figures 6, 7). All measurements were recorded using the HOBO® Micro Station Data Logger.

### Data Analysis

A two-way ANOVA analysis was used with an RCBD design to analyze the main effects of the watering treatments and the seasonal and combined effects of both the watering treatments and seasons on SWC, soil pH, soil elemental composition, and the composition of fungal and bacterial communities. As multivariate analysis takes all microbial community variables into consideration, we employed multivariate PCA ordination analysis to discriminate the patterns of the water-treated samples in the microbial community variables during both seasons.

The significance of PCA scores was confirmed by two-way PERMANOVA using the Euclidean distance, to learn the analytic differences between the water treatment groups (CK, T1, T2, and T3). Pearson correlation analysis was performed to find the correlations between microbial phyla and soil chemical variables. Canonical correspondence analysis (CCA) was performed to analyze the correlations between the microbial characteristics and soil chemical variables of two seasons.

Indicator species analysis (ISA) was conducted with R software using the IndVal script in the labdsv package (Roberts, 2016) and employed to identify microbial taxa that existed in the majority of groups of one season but were absent in the majority of groups of the other season. ISA calculates an indicator value (IV, %) as mentioned in Dufrêne and Legendre (1997). The IV is a key measure of the relative average abundance of a species or OTUs in a cluster. The maximum IV is 100 when an OTU is present in all samples of one season group or one water treatment group. To verify the significance test of the IV, the *p* values were measured with 100 iterations, where the different group samples were arbitrarily marked and an IV calculated in each iteration. The *p* values for the IV measurement were selected for multiple comparisons using the false discovery rate correction.

A one-way ANOVA was performed using SAS version 9.0 and determined the effect of water-addition treatments using five replications and four treatments. We employed Duncan’s multiple range test (MRT) with least significant difference (LSD; *p* < 0.05) to identify the significant difference for mean comparison. All numerical and graphical data were analyzed using SPSS version 19.0 (SPSS Inc., Chicago, IL, United States), Microsoft Excel 2007 (Microsoft Press, Redmond, WA, United States), Sigma Plot version 10.0 (Systat software, Inc., Richmond, CA, United States), and PAST 4.03 (Hammer et al., 2001).

## Results

### Effects of Water Addition on Elemental Compositions of Soils

**FIGURE 1:**
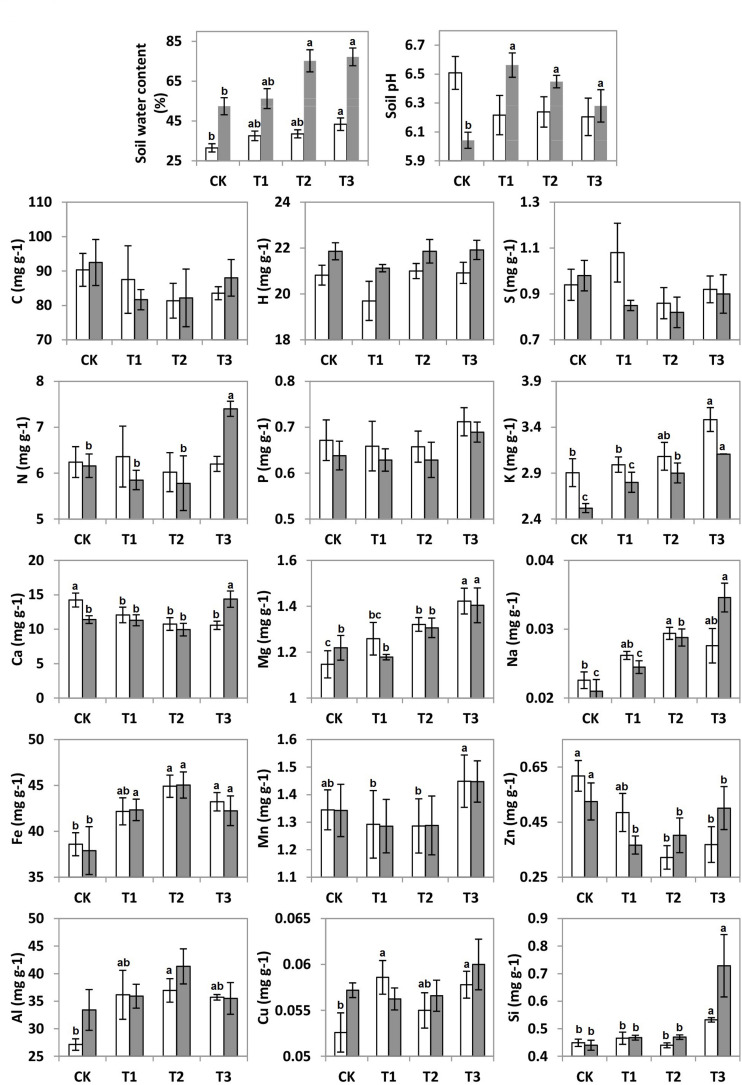
Soil water content (SWC), soil pH, and soil elements C, H, N, S, P, K, Ca, Mg, Na, Al, Fe, Mn, Zn, Cu, and Si concentrations (mg g^–1^) of 20 plots during the dry (white bars) and wet (gray bars) seasons under 0% (CK), +20% (T1), +40% (T2), and +60% (T3) watering treatments. The different letters (a, b and c) indicated significant difference between watering treatments detected by Duncan’s multiple range test (*p* < 0.05).

It can be seen that SWC revealed an increasing trend with the further addition of water for both seasons (Figure 1). During the wet season, the SWC was 1.7-fold higher than that in the dry season (*F* = 6.92; *p* < 0.01; Table 1). Compared to the CK, the soil pH during the wet season increased significantly in the water-treated plots, whereas no significant changes were observed during the dry season (*F* = 8.31; *p* < 0.01; Table 1).

**TABLE 1:** Watering treatments had significant effects on the soil pH, soil water content, and soil elemental composition during the dry and wet seasons.

Soil variables	*F* and *p* values	
	Dry season	Wet season
pH	1.42 (0.27)	8.31 (**<0.01**)
SWC	3.93 (**0.03**)	6.92 (**<0.01**)
N	0.10 (0.96)	4.62 (**0.02**)
C	0.43 (0.73)	0.70 (0.57)
H	1.21 (0.34)	0.95 (0.44)
S	1.20 (0.34)	1.20 (0.34)
Fe	4.59 (**0.02**)	5.91 (**0.01**)
P	0.37 (0.78)	0.96 (0.44)
K	3.74 (**0.03**)	9.16 (**<0.01**)
Ca	3.26 (**0.05**)	6.21 (**0.01**)
Mg	4.22 (**0.02**)	3.79 (0.03)
Mn	0.58 (0.64)	0.65 (0.6)
Zn	5.03 (**0.01**)	5.57 (**0.01**)
Cu	2.19 (0.13)	0.93 (0.45)
Na	3.77 (**0.03**)	14.24 (**<0.01**)
Al	3.31 (0.05)	1.24 (0.33)
Si	8.68 (**<0.01**)	5.53 (**0.01**)

*The values in bold indicate statistical significance at *p* < 0.05 in one-way ANOVAs.*

The addition of water significantly altered the elemental compositions of the karst soils during both the dry and wet seasons (*p* < 0.05). Between the 15 elements, the soil concentrations of K, Ca, Mg, Na, Fe, Al, Zn, and Si varied considerably across the water treatment plots (*p* < 0.05), while no significant changes were observed for the other elements (Figure 1). The non-metal soil elements (C, H, N, S, and P, except for Si) were relatively constant with water addition; however, the metal elements (Al, Na, Mg, Fe, and K, along with Si) increased significantly, whereas Zn and Ca decreased (Table 1).

There were seasonal changes in the elemental composition of the treated soils. During the wet season, the H concentration of the soil was approximately 5.2% higher than during the dry season (Table 1). For both seasons, the soil Na and K in the water-treated plots increased significantly in contrast to that in the CK (Figure 1). Compared to the CK, the soil Ca decreased significantly in the water-treated plots during the dry and wet season (Table 1).

**FIGURE 2:**
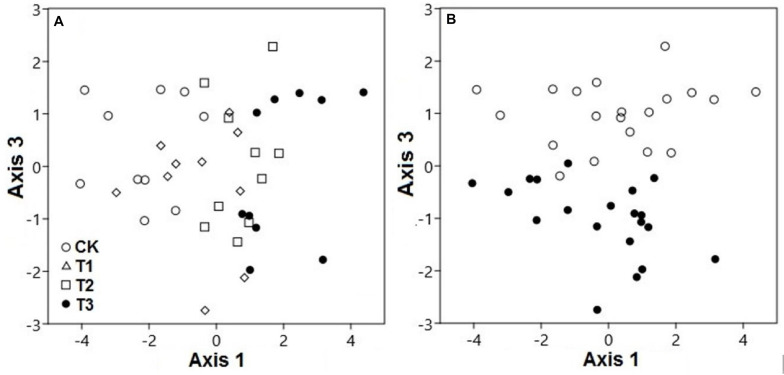
Principal component analysis demonstrated the soil elemental variables of two seasons under 0% (CK), +20% (T1), +40% (T2), and +60% (T3) watering treatments. The elemental variables of soils were compositionally distinct between **(A)** watering treatments along axis 3 and **(B)** seasons along axis 1. The watering treatments are indicated by different colors and geometric shapes (white circles, CK; white squares, T1; white triangles, T2; and black circles, T3), whereas the seasons are indicated by different colors (white circles, CK; black circles, T3).

Based on PCA, both water treatments and seasons had considerable interactive effects on the SWC, pH, and elemental concentrations (Figure 2). The PCA scores showed that the elemental variables in the soil differed significantly, contingent on the watering treatment at axis 1 (explaining 34.4% variation; *pseudo-F* = 19.6; *p* < 0.001) and season at axis 3 (explaining 14.9% variation; *pseudo-F* = 95.9; *p* < 0.001). Axis 2 was discounted for the differences of watering samples and results were confirmed by two-way PEMANOVA analysis (Supplementary Table 1 and Supplementary Figure 8). This indicated that the watering treatment was the primary factor, while the season was secondary in terms of affecting the elemental composition of the soil.

For both seasons, the elemental composition of the soil in the water-treated plots differed from the CK plots (Figure 2A). Season also played a substantial role in the compositional variation of soil elements (*p* < 0.01). The elemental variables of the soil during the dry season, with relatively low moisture, were quite distinct from those under high moisture during the wet season, along axis 3 (Figure 2B).

### Effects of Water Addition on Microbial Diversity

**FIGURE 3:**
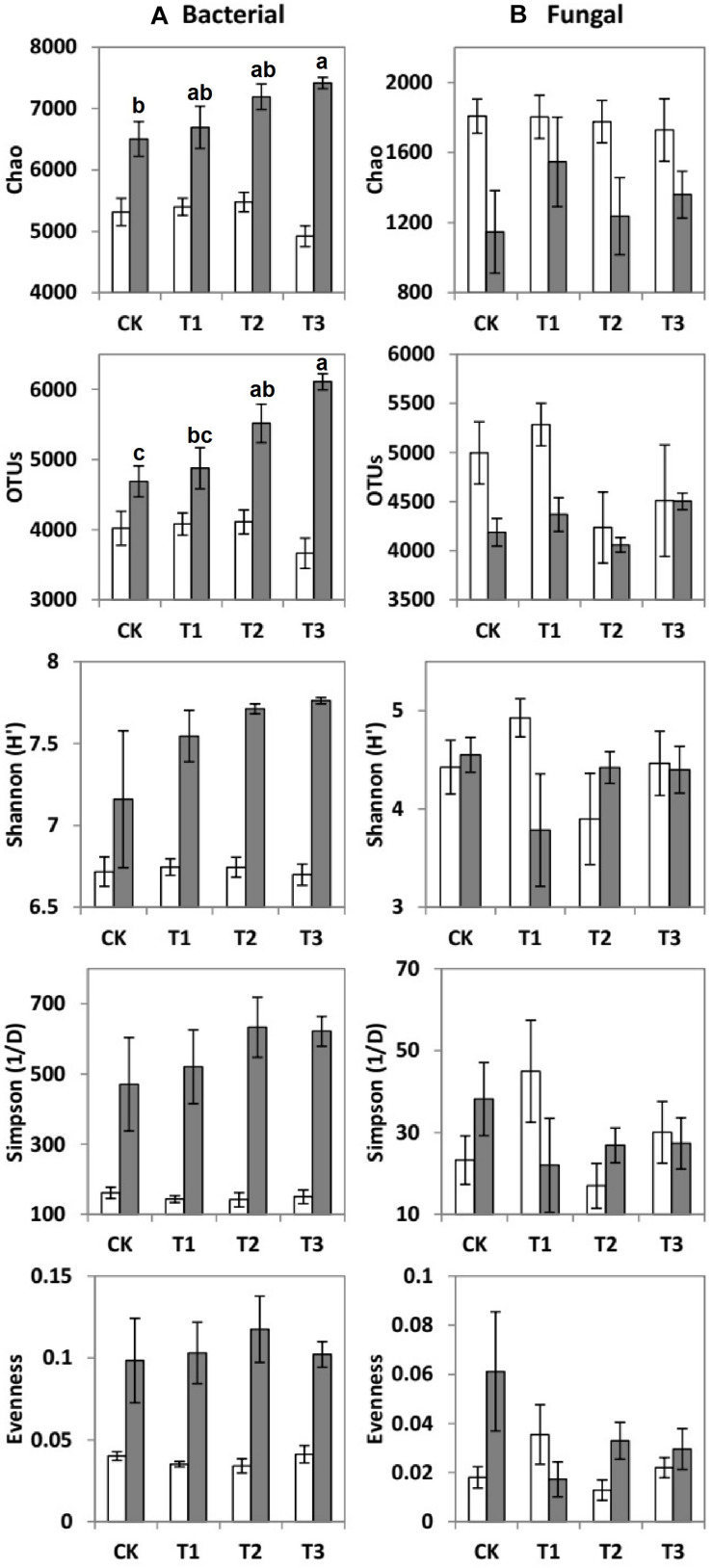
Means ± SE of **(A)** bacterial and **(B)** fungal community richness and diversity indices during the dry (white bars) and wet (gray bars) seasons under 0% (CK), +20% (T1), +40% (T2), and +60% (T3) watering treatments. The different letters (a, b and c) indicated significant difference between watering treatments detected by Duncan’s multiple range test (*p* < 0.05).

Compared to the CK, the bacterial OTUs (*F* = 7.51; *p* < 0.01) during the wet season were significantly decreased in the water-treated plots, whereas no significant changes were observed during the dry season (Table 2). Conversely, the fungal evenness during the dry season was higher in the water-treated plots during the dry season, whereas no changes were observed during the wet season (Figure 3).

**TABLE 2:** Watering treatment had significant effects on the microbial community richness and diversity indices during the dry and wet seasons.

Microbial community	Community richness and diversity indices	*F* and *p* values	
		Dry season	Wet season
Bacterial community	OTUs	1.07 (0.39)	**7.51 (<0.01)**
	Chao	1.98 (0.16)	2.87 (0.07)
	Shannon (H)	0.11 (0.95)	1.48 (0.26)
	Evenness	0.88 (0.47)	0.19 (0.90)
	Simpson (I/D)	0.28 (0.84)	0.66 (0.59)
Fungal community	OTUs	1.48 (0.26)	2.47 (0.10)
	Chao	0.08 (0.97)	0.64 (0.60)
	Shannon (H)	1.63 (0.22)	1.07 (0.39)
	Evenness	1.88 (0.17)	1.80 (0.19)
	Simpson (I/D)	2.09 (0.14)	0.69 (0.57)

*The values in bold indicate statistical significance at *p* < 0.05 in one-way ANOVAs.*

The values of specific OTUs and diversity indices [Shannon (H′), Simpson (1/D), Evenness, and Chao 1 estimator] of the bacterial communities were all considerably higher during the wet season than during the dry season (Figure 3A). However, the Chao 1 estimator of fungal communities was significantly higher during the dry season than during the wet season (Figure 3B).

### Effect of Water Addition on Microbial Community Composition

During the dry season, the microbial phyla did not show any significant changes in response to the addition of water (Supplementary Table 2). However, during the wet season, the relative abundance of Acidobacteria, Planctomycetes, and Verrucomicrobia decreased by 1.25, 5.88, and 64.7% at T1; 22.5, 17.6, and 35.3% at T2; and 29.6, 52.9, and 82.4% at T3, respectively, compared with the CK (Table 3 and Supplementary Table 2). In contrast to the CK, the relative abundance of Actinobacteria increased significantly by 27.9, 11.5, and 21.0% at T1, T2, and T3, respectively.

**TABLE 3:** Watering treatments had significant effects on the average relative abundance of microbial phyla during the dry and wet seasons.

Main phyla of microbial community	*F* and *p* values	
	Dry season	Wet season
Actinobacteria	0.33 (0.81)	**6.03 (0.01)**
Proteobacteria	2.36 (0.11)	3.02 (0.06)
Acidobacteria	0.14 (0.93)	**3.44 (0.04)**
Chloroflexi	1.55 (0.24)	1.55 (0.24)
Planctomycetes	0.08 (0.97)	**3.39 (0.04)**
Nitrospirae	0.02 (0.99)	1.10 (0.38)
Bacteroidetes	0.23 (0.88)	1.88 (0.17)
Firmicutes	0.51 (0.68)	0.42 (0.74)
Verrucomicrobia	0.12 (0.95)	**6.98 (<0.01)**
Ascomycota	0.41 (0.75)	0.45 (0.72)
Basidiomycota	0.11 (0.95)	1.28 (0.31)
Mortierellomycota	1.85 (0.18)	0.64 (0.60)
Chytridiomycota	1.96 (0.16)	0.48 (0.70)
Glomeromycota	1.62 (0.22)	0.99 (0.42)

*The values in bold indicate statistical significance at *p* < 0.05 in one-way ANOVAs.*

**FIGURE 4:**
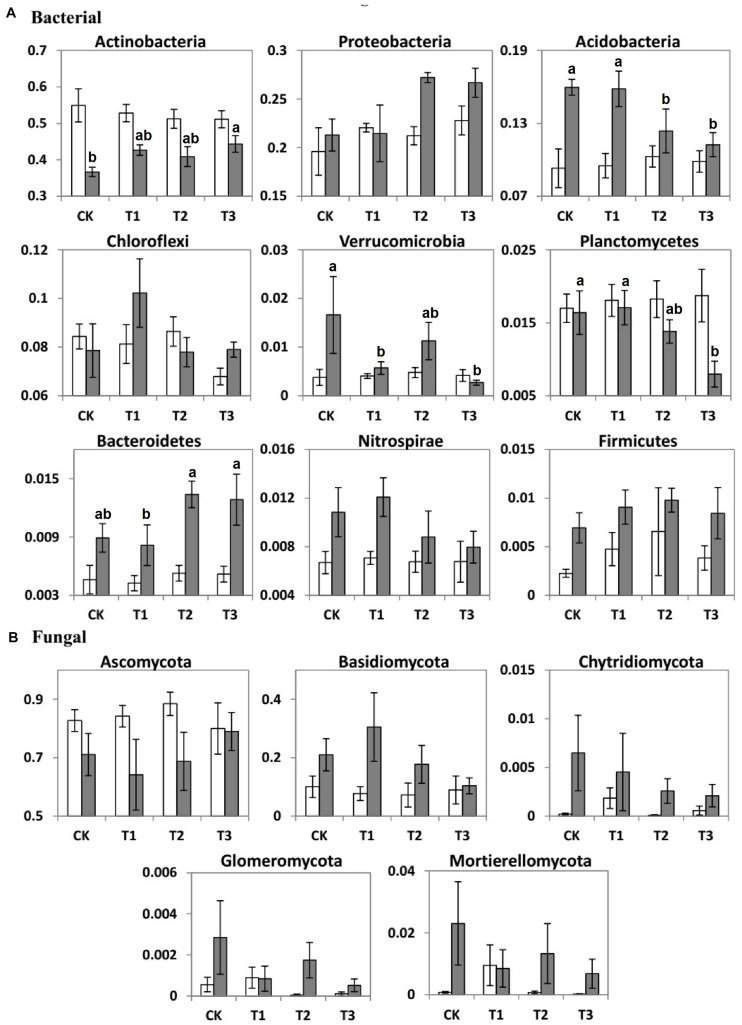
Bar charts showing the average relative abundance (%) of important **(A)** bacterial and **(B)** fungal phyla of the two seasons (dry, white bars; wet, gray bars) under 0% (CK), +20% (T1), +40% (T2), and +60% (T3) watering treatments. The different letters (a, b) indicated significant difference between watering treatments detected by Duncan’s multiple range test (*p* < 0.05).

The seasons had significant impacts on the average relative abundance of bacterial and fungal communities (Table 3). Compared to the dry season, the average relative abundance of Proteobacteria, Acidobacteria, Chloroflexi, Nitrospirae, Bacteroidetes, Firmicutes, and Verrucomicrobia were considerably higher during the wet season (Figure 4A). In contrast, Actinobacteria and Planctomycetes exhibited a higher average relative abundance during the dry season (*p* < 0.05). The average relative abundance of some fungal phyla was considerably higher during the wet season as compared to the dry season, i.e., Basidiomycota, Chytridiomycota, and Mortierellomycota (Figure 4B). On the contrary, the average relative abundance of phylum Ascomycota was significantly higher during the dry season (Figure 4).

Based on ISA, we discovered that Actinobacteria, Proteobacteria, Bacteroidetes, and Ascomycota were the dominant microbial phyla during the dry season (Tables 4, 5). The IV of *Phytohabitans*, *Pilimelia*, *Arthrobacter*, and *Non-omuraea* (Actinobacteria), *Inquilinus* and *Rhodovastum* (Proteobacteria), *Sediminibacterium* (Bacteroidetes), and *Leptogium*, *Cladosporium*, *Neodevriesia*, *Toninia*, *Catenulostroma*, *Phyllosticta*, *Paraconiothyrium*, *Beauveria*, *Medicopsis*, *Cyberlindnera*, *Thaxteriellopsis*, and *Monilinia* (Ascomycota) were >70% (Tables 4, 5). Compared to the CK, only one fungal genus, i.e., *Thaxteriellopsis*, of the microbial community was significantly changed in all water-treated groups (T1, T2, and T3) with an IV of 89.4% (Supplementary Tables 3, 4).

**TABLE 4:** Indicator species analysis showing that bacterial genera are significantly associated with dry and wet seasons in a degraded karst area of Southwestern China.

Bacterial phyla	Bacterial genera	Indicator value (%)	*p* value
**Dry season**			
Actinobacteria	*Phytohabitans*	99.3	0.001***
	*Pilimelia*	98.8	0.001***
	*Arthrobacter*	91.3	0.001***
	*Non-omuraea*	91.3	0.001***
Proteobacteria	*Inquilinus*	91.2	0.001***
	*Rhodovastum*	84.2	0.001***
Bacteroidetes	*Sediminibacterium*	77.9	0.001***
	*Rhodocytophaga*	59.3	0.026*
**Wet season**			
Acidobacteria	*Terriglobus*	58.9	0.012*
Actinobacteria	*Micromonospora*	99.5	0.001***
	*Kibdelosporangium*	98.3	0.001***
	*Iamia*	96.4	0.001***
Chloroflexi	*Roseiflexus*	99.9	0.001***
Firmicutes	*Paenibacillus*	80.7	0.003**
Planctomycetes	*Isosphaera*	79.1	0.001***
Proteobacteria	*Bradyrhizobium*	99.7	0.001***
	*Craurococcus*	99.4	0.001***
	*Bosea*	96.3	0.001***
	*Phaselicystis*	90.9	0.001***
	*Acinetobacter*	90.7	0.038*
	*Ensifer*	89.9	0.001***
	*Pseudomonas*	88.1	0.001***
	*Cupriavidus*	86.2	0.001***
	*Dokdonella*	85.9	0.001***
	*Burkholderia*	83.2	0.006**
	*Aquicella*	82.9	0.001***
	*Novosphingobium*	76.7	0.001***
	*Rickettsiella*	65.2	0.009**
	*Haliangium*	98.8	0.001***
Verrucomicrobia	*Chthoniobacter*	88.0	0.001***

*Indicates significance (*p* < 0.05). **Indicates high significance (*p* < 0.01). ***Indicates very high significance (*p* < 0.001).

**TABLE 5:** Indicator species analysis showing that fungal genera are significantly associated with dry and wet seasons in a degraded karst area of Southwestern China.

Fungal phyla	Fungal genera	Indicator value (%)	*p* value
**Dry season**			
Ascomycota	*Leptogium*	94.0	0.003**
	*Cladosporium*	92.8	0.001***
	*Neodevriesia*	91.3	0.002**
	*Toninia*	83.7	0.001***
	*Catenulostroma*	82.1	0.009**
	*Phyllosticta*	79.8	0.005**
	*Paraconiothyrium*	78.0	0.011*
	*Beauveria*	75.7	0.001***
	*Medicopsis*	75.6	0.014*
	*Cyberlindnera*	71.8	0.010**
	*Thaxteriellopsis*	71.7	0.005**
	*Monilinia*	71.4	0.010**
	*Arthropsis*	68.2	0.009**
	*Magnaporthiopsis*	62.8	0.013*
	*Heterodermia*	62.6	0.028*
	*Pseudocercospora*	60.6	0.013*
	*Pyrenula*	59.4	0.014*
	*Satchmopsis*	52.4	0.030*
**Wet season**			
Ascomycota	*Dialonectria*	61.6	0.014*
	*Verruconis*	57.1	0.042*
	*Vermiconia*	54.7	0.031*
Basidiomycota	*Geminibasidium*	79.2	0.013*
	*Entoloma*	62.1	0.012*
Chlorophyta	*Trebouxia*	91.3	0.031*
Chytridiomycota	*Spizellomyces*	74.2	0.022*

*Indicates significance (*p* < 0.05). **Indicates high significance (*p* < 0.01). ***Indicates very high significance (*p* < 0.001).

During the wet season, Actinobacteria, Proteobacteria, Acidobacteria, Chloroflexi, Planctomycetes, Firmicutes, Verrucomicrobia, Ascomycota, Basidiomycota, Chlorophyta, and Chytridiomycota were the key microbial phyla across all plots. The genera *Micromonospora*, *Kibdelosporangium*, and *Iamia* (Actinobacteria), *Roseiflexus* (Chloroflexi), *Paenibacillus* (Firmicutes), *Bradyrhizobium*, *Craurococcus*, *Bosea*, *Phaselicystis*, *Acinetobacter*, *Ensifer*, *Pseudomonas*, *Cupriavidus*, *Dokdonella*, *Burkholderia*, *Aquicella*, *Haliangium*, and *Chthoniobacter* (Proteobacteria), and *Trebouxia* (Chlorophyta) had IV of more than 80%.

The IV of *Novosphingobium* (Proteobacteria), *Quadrisphaera* and *Sphaerisporangium* (Actinobacteria), *Petrakia* (Ascomycota), and *Trebouxia* (Chlorophyta) was 88.4, 85.6, 84.4, 85.6, and 98.8% in all water-treated samples (T1, T2, and T3), respectively (Supplementary Tables 3, 4).

**FIGURE 5:**
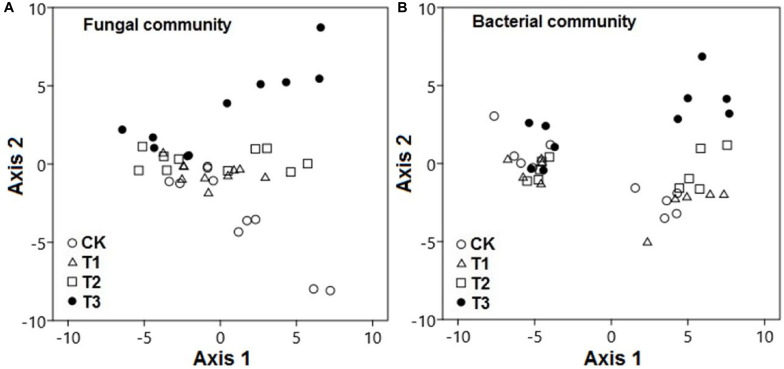
Microbial communities were significantly distinct between watering treatments. Principal component analysis demonstrated the **(A)** fungal and **(B)** bacterial community compositions in response to increased precipitation. The watering treatments are indicated by different colors and geometric shapes (white circles, CK; white squares, T1; white triangles, T2; and black circles, T3). Each point represents a specific community in one of the treatment plots or control plots. Points that are close together are more similar to one another than points that are far apart.

The PCA results revealed that the watering treatments had potent effects on the composition of fungal and bacterial communities. The composition of fungal communities in the water-treated samples was significantly different from that in the CK samples along axis 2 (Figure 5A; pseudo-*F* = 51.2; *p* < 0.001). Likewise, the composition of bacterial communities in the water-treated samples was significantly distinct from that in the CK samples along axis 2 (Figure 5B; pseudo-*F* = 22.8; *p* < 0.001).

During the wet season, compositions of bacterial and fungal communities in the water-treated samples were quite distinct from those in the CK samples along axis 2; however, this was not the case during the dry season (*p* < 0.001).

**FIGURE 6:**
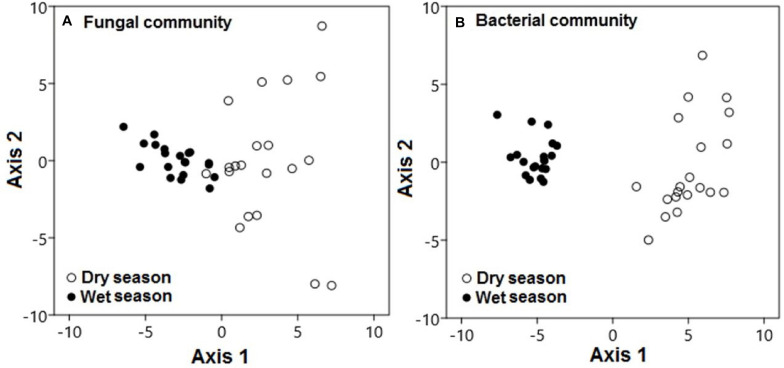
Microbial communities were significantly distinct during the dry and wet seasons across all treatments. Principal component analysis demonstrated **(A)** fungal and **(B)** bacterial community compositions during the dry (black circles) and wet (white circles) seasons. Each point represents a specific community during either the dry or wet season. Points that are close together are more similar to one another than points that are far apart.

Seasonal conditions also played specific roles in the modification of microbial community compositions in this karst habitat. During the dry season, fungal communities were significantly distinct from those during the wet season along axis 1 (Figure 6A; pseudo-*F* = 102.62; *p* < 0.001). This pattern also held for the composition of bacterial communities, which varied between the dry and wet seasons along axis 1 (pseudo-*F* = 674.7; *p* < 0.001; Figure 6B).

### Relationship Between Microbial Community Structures and Elemental Concentrations in Soils

**FIGURE 7:**
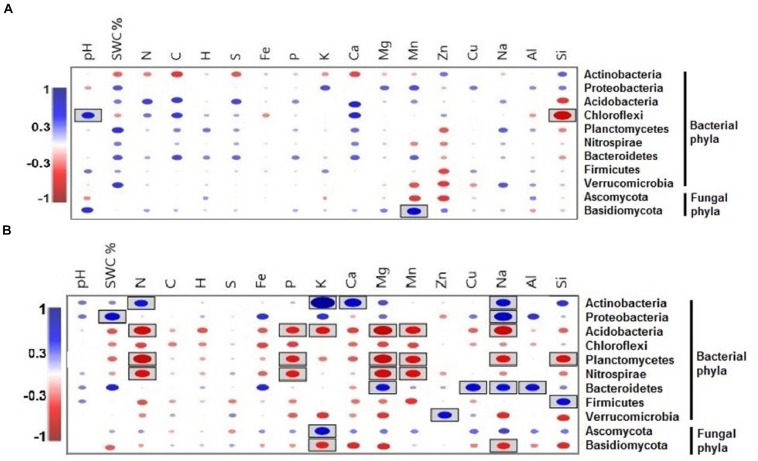
Pearson correlation coefficients between important soil microbial phyla and soil environmental variables of the two seasons, **(A)** dry season and **(B)** wet season across all the treatments. Blue indicates a positive correlation, and red refers to a negative correlation. The statistically significant values are boxed (*p* < 0.05).

Only the phylum Chlorflexi exhibited a significantly positive correlation with pH and a negative correlation with Si during the dry season (Figure 7A), whereas the relative abundance of the phylum Basidiomycota had a significantly positive correlation with Mn. Between all of the soil elements, C, H, S, and Fe did not show any significant correlation with microbial phyla during the wet season (Figure 7B).

**FIGURE 8:**
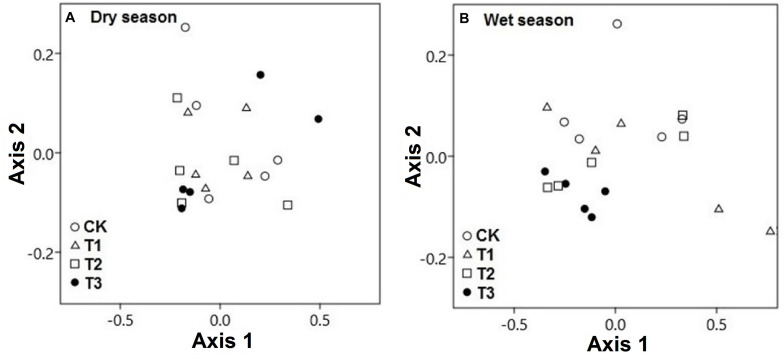
Canonical correspondence analysis (CCA) demonstrated the soil microbial phyla and soil multielement variables of the two seasons, **(A)** dry season and **(B)** wet season under 0% (CK), +20% (T1), +40% (T2), and +60% (T3) watering treatments. The watering treatments are indicated by different colors and geometric shapes (white circles, CK; white squares, T1; white triangles, T2; and black circles, T3). Each point represents a specific community in one of the water treatment or control plots. Points that are close together are more similar to one another than points that are far apart.

**FIGURE 9:**
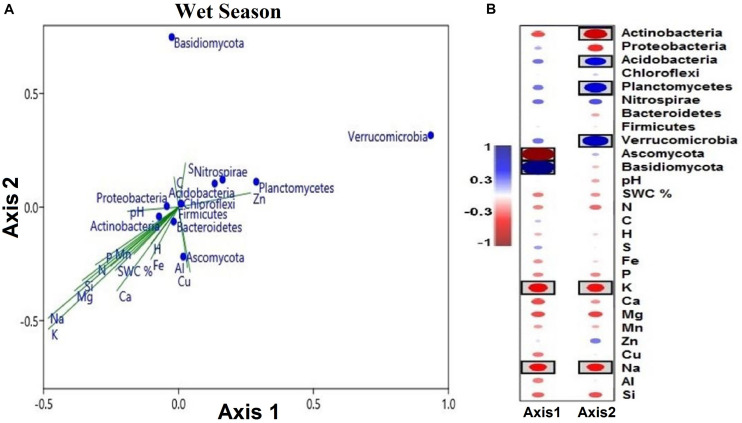
**(A)** Soil microbial phyla defined by the two variables (axes 1 and 2) extracted from canonical correspondence analysis (CCA), based on multielement composition during the wet season. Solid symbols represent the mean of each phylum. The position of blue circles in a plot relative to the direction of green lines approximates correlations between microbial phyla and the gradient of element concentrations. The lengths of green lines indicate the overall contribution of the element to the analysis. The directions of the green lines indicate the element correlation with each axis (vector lines parallel to an axis are highly correlated with that axis). Angles between the vector lines show correlations between elements. **(B)** Correlation coefficients of soil microbial phyla and soil variables with canonical axes (axes 1 and 2). Blue indicates a positive correlation, and red refers to a negative correlation. The statistical significant values are boxed (*p* < 0.05).

The CCA of both seasonal studies explained the composition of soil microbial communities on the basis of multielement composition under different water treatment regimens. During the wet season, the composition of microbial communities in the water-treated samples was appreciably distinct from that in the CK samples along axis 2 but not during the dry season (Figure 8A). The CCA results revealed that the CK samples were clearly distinct from water-treated samples during the wet season, along axis 2 (*pseudo-F* = 3.91; *p* = 0.02; Figure 8B). The first two axes, axes 1 and 2, explained the majority of variation in the elemental composition among the microbial phyla (Figure 9 and Supplementary Table 5).

The segregation of the microbial phyla is represented graphically in the biplots of axes 1 and 2. Axis-2 of CCA (explaining 7.98% of the variation) was primarily loaded with soil microbial phyla and soil multielement variables and significant negatively correlated with soil K and Na (*p* < 0.05; Figure 9A). As canonical correlations with axis 2 were significantly correlated with K and Na across microbial communities, K and Na were the most effective in distinguishing between microbial phyla (Figure 9B).

## Discussion

Changes in precipitation due to climate change, initiate shifts in the compositions, and diversity of soil microbial communities have been widely reported (Cregger et al., 2012; Zhang et al., 2016). To the best of our knowledge, our study is the first effort to characterize the influence of water addition on the diversity and community composition of microbes in soil and its elemental composition in a karst ecosystem. In the context of climate change, our findings have significant implications for understanding the consequences of altering the structure and diversity of microbial communities and elemental compositions of soils due to increased precipitation in such karst ecosystems.

### Water Addition Altered Elemental Concentrations of Karst Soils During Both Dry and Wet Seasons

Our results clearly indicated that the addition of water had a significant effect on the elemental concentrations of soils during both the dry and wet seasons in a degraded karst community. Further, the elemental concentrations of soils differed considerably between the two seasons (Table 1). Interestingly, for both seasons, the non-metal soil elements (C, H, N, S, and P, except for Si) were relatively stable with water addition; however, the metal elements (Al, Na, Mg, Fe, Cu, and K, along with Si) increased significantly, whereas Zn and Ca decreased. This was consistent with a study of Wang et al. (2016), which reported that water addition led to decreased concentrations of most elements (e.g., Ca^+2^, Mg^+2^, SO^+2^, and HCO_3_^–^, with the exception of K^+^ and Na^+^) in the karst areas of Southwestern China. Such patterns in elemental variations might characterize the chemical weathering of karst silicate rocks (granites and metamorphic rocks) due to water addition, whereby one of the key natural processes is the release of K^+^ and Na^+^ into the ambient environment (Lyu et al., 2018). Similarly, such water addition and elemental concentration patterns in karst soils have been observed in other geologically derived soils. In estuarine wetland ecosystems, soil C, N, and P had no obvious change with flooding intensity but differed significantly with soil depth (Wang et al., 2018). Under 2 years of watering treatments, no significant effects were observed for soil extractable N and P in tropical forests (Wang et al., 2019). However, in a greenhouse experiment, Misra and Tyler (1999) reported that with increased soil moisture, soil P, Mn, and K increased along with pH and HCO_3_^–^, while Ca, Mg, and Zn decreased.

In terms of mechanisms, variations in the elemental concentrations of soils are instigated by multiple factors, with the predominant one being soil moisture in karst soils. In this context, the key processes involved in the release and fixation of soil elements include precipitation–dissolution and desorption–adsorption (Singh and Schulze, 2015). The elements of soil resident solutions are partially altered against the changes in soil moisture by the precipitated, exchangeable, or adsorbed zones in the solid phases of the soil (Wolt, 1994). Increases in soil moisture were observed to alter their ionic concentrations, distribution, and formation of complex structures (Fotovat and Naidu, 1998). Karst soils are rich in Ca and Mg due to the presence of carbonate rocks such as calcite and dolomite (Yuan et al., 2017), where their concentrations are interactive (Li et al., 2005). Verily, soil-resident Ca in karst areas is typically regulated by precipitation and calcite dissolution (Buhmann and Dreybrodt, 1985). However, these authors do not consider the dissolution of CaCO_3_ in high moisture calcareous soils as a key factor in determining HCO_3_^–^, which is clearly evident from the decline in Ca concentrations in the soil solution (Misra and Tyler, 1999; Misra, 2003). As a consequence, low concentrations of Zn are found in the soil solution when the moisture is increased, which results in the lower availability of Zn by precipitation of franklinite-like solid material (Sajwan and Lindsay, 1986). Actually, the concentration of Zn in the soil solution depends on factors such as concentrations of HCO_3_^–^ and macronutrients (Misra and Tyler, 1999; Misra, 2003). In fact, decreases in the concentrations of karst rock-derived nutrients in soils, due to water addition, suggest that the effects of increased precipitation on leaching exceed its impacts on weathering and deposition at wetter sites. It has been well documented in previous studies that the availability of K in soil increases with higher soil moisture (Misra and Tyler, 1999; Zeng and Brown, 2000; Singh and Singh, 2004).

### Water Addition Alters Soil Microbial Community Structures Through Changes in Soil Conditions

Our results clearly revealed that the relative abundance of Acidobacteria, Plantomycetes, and Verrucomicrobia were negatively correlated with soil K and Na content, while Actinobacteria had a significantly positive correlation with K^+^ (*r*^2^ = 0.70, *p* < 0.001) and Na^+^ (*r*^2^ = 0.36, *p* < 0.01) contents (Supplementary Table 6) during the wet season, and the addition of water did not influence fungal phyla. This suggests that the karst bacterial communities can be influenced by the addition of water during the wet season likely linked to changes in soil K and Na contents. A similar phenomenon was observed in karst farmland soils in Central China (Yun et al., 2016). Results showed that the relative abundance of Actinobacteria had significant positive correlation with soil K^+^ content (*r*^2^ = 0.28, *p* < 0.01). In fact, the effects of K and Na on soil microbes were widely observed in previous studies. For instance, the addition of K and Na enhanced the mass production of the cellulase enzymes that are required to break down C-rich macromolecules by microbes (Kaspari et al., 2008, 2009). In fact, the availability of K was positively associated with litter decomposition in ecosystems (Tripathi, 1992; Laskowski et al., 1995; Pandey et al., 2007), and Na benefitted plant consumer metabolism in both herbivores and decomposers (Frausto da Silva and Williams, 2001; Kaspari et al., 2009).

**FIGURE 10:**
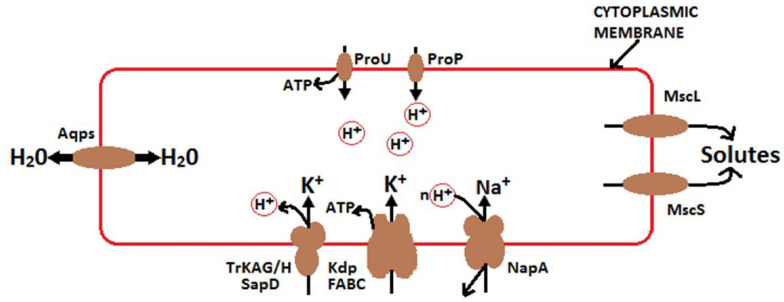
Mechanisms of osmoregulation and energy generation in bacteria modified from Wood (2015). Under high osmotic pressure, solutes transport from the soil solution and accumulate in bacteria. K^+^ –H + symporter P type ATPase Kdp (Los and Murata, 2000) and TrkAG/H SapD (Harms et al., 2001) stimulate the uptake of K+. K + diffusion suppresses the effect of ΔΨ. ProP is a proton symporter and a member of the major facilitator superfamily. ProP releases H+ in the cytoplasm of bacterial cell, leading to the acidification of the cytoplasm. ProU generates ATP and is utilized for protein synthesis and other functions. ProP and ProU are similarly broad in substrate specificity (Wood, 2015). The Na+/H+ antiporter NaPA mediates Na+ uptake under acidic conditions (Uzdavinys et al., 2017). Mechanosensitive channels, including MscL and MscS, relay solutes from the cytoplasm of osmotically down-shocked bacteria. Aqps suppresses the effect of osmotic pressure (π) by accelerating the transmembrane water flux.

It is well recognized that high concentrations of soluble salts (particularly K^+^ and Na^+^) affect microbes via osmotic effects. Various studies have reported that bacterial osmoregulation processes are controlled by transporters, enzymes, and channels that mediate solute accumulation and release (Wood, 2011, 2015). Bacteria are externally enclosed by selectively permeable cytoplasmic membranes, which often include aquaporins (Figure 10). They respond to changes in external osmotic pressure by releasing or accumulating solutes (often K^+^), thereby transducing water fluxes. Under extreme conditions, some halotolerant species increase KCl concentrations within cells, and their proteins work only in high saline conditions (Wood, 2015). In fact, these species have adapted to tolerate high osmoregulatory solute accumulation. Osmoregulatory solutes accumulate through active transport or synthesis when the osmotic pressure increases and releases via mechanosensitive channels, when the osmotic pressure decreases (Wood, 2011, 2015). Moreover, osmoregulatory systems are transcriptionally controlled and mediated by small regulatory RNA (a key determinant of cell walls), which may affect osmoregulatory systems (Altendorf et al., 2009; Krämer, 2010). According to Wood (2015), the accumulation of soluble solutes in cells strongly activate the growth of bacteria at high external osmotic pressures and solute-releasing channels discharge solutes to survive osmotic down-shocks. As a result, the growth rate of bacterial populations is directly proportional to cellular hydration, where this accumulation of soluble solutes influences population growth and cytoplasmic rehydration.

Additionally, we used ISA to identify specific members linked to dominant phyla, such as Actinbacteria, Acidobacteria, Planctomycetes, and Verrucomicrobia, which showed a significant correlation with content of Na^+^ and K^+^. The main drivers for the reduction or abundance of these phyla were *Micromonospora*, *Kibdelosporangium*, and *Iamia* (Actinobacteria), *Terriglobus* (Acidobacteria), *Isosphaera* (Planctomycetes), and *Chthoniobacter* (Verrucomicrobia; Table 4). In our study, an increase in relative abundance of dominant Actinobacteria (Gram-positive bacteria) was primarily associated with the presence of halo-tolerant bacteria, such as *Micromonspora* (Ballav et al., 2015), *Iamia* (Plotnikova et al., 2011; Ma and Gong, 2013), and *Kibdelosporangium* (Qin et al., 2018). Abdulla (2009) found that *Kibdelosporangium* and *Nocardioides* were potential members of Actinomycetes, which played multiple roles in rock weathering, such as metal leaching, acid production, and the solubilization of phosphate and sulfate.

Moreover, Gram-positive bacteria and archaea can better tolerate high matric potential than Gram-negative bacteria due to their stronger cell walls (Fierer et al., 2003; Ma et al., 2015). Additionally, Gram-positive bacteria can accumulate compatible solutes to assist in maintaining high turgor, which requires an adjustment in the regulation of osmoprotectant uptake and integration of different systems in cells. Likewise, archaea, which are often found in high-salt as well as high-temperature environments, use the same general strategies for osmoadaptation as eubacterial and eukaryotic organisms. Under optimal growth conditions, most of the archaea examined have high intracellular concentrations of inorganic cations, mainly K^+^. To cope with the high intracellular concentrations of K^+^, many archaea have evolved proteins that are exceedingly rich in acidic amino acids (glutamic acid and aspartic acid) compared to basic amino acid (arginine and lysine) residues (Jarrell et al., 1984). In addition to these large excess of negatively charged amino acid residues, the hydrophobicity of the proteins of halophilic archaea is reduced. This in turn reduces the salting-out effects of K^+^ and helps the protein to maintain its strength under high saline conditions (Martin et al., 1999).

### Seasonal Variations in Microbial Composition and Diversity

In regions with two distinct seasons, the composition of soil microbial communities varied substantially across seasons (Waldrop and Firestone, 2006; Cregger et al., 2012; Taketani et al., 2017). Our results highlighted variations in the composition and diversity of soil microbial communities in degraded karst areas between two distinct seasons.

First, based on our PCA results, during the dry season, bacterial and fungal communities were clearly distinct from the communities during the wet season (Figure 6). It was shown that soil microbial communities that live in dry soils become active and successively shift their compositions during the wet season in the Mediterranean and semiarid ecosystems (Kavamura et al., 2013; Barnard et al., 2015; Taketani et al., 2017). Seasonal variations in rainfall play a considerable role in determining the composition of microbial communities in a semiarid woodland ecosystems (Cregger et al., 2012). Furthermore, Bell et al. (2014) suggested that a 25% increase in seasonal rainfall in a desert grassland may alter the microbial community structure.

Second, our results clearly revealed that OTUs and diversity indices in bacterial communities during the wet season were significantly higher than those during the dry season; however, this was not the case for fungi (Figure 2). Similar reports were previously published for the bacterial communities of soils in Caatinga, Brazil (Kavamura et al., 2013; Taketani et al., 2017), and Californian grasslands (Barnard et al., 2013, 2015). In fact, the decrease in moisture triggered several changes within the soil that directly affected its bacterial community diversity, such as the decreased availability of nutrients (Placella et al., 2012), low pore connectivity (Carson et al., 2010), and increased O_2_ availability (Silver et al., 1999).

These changes resulted in a higher bacterial abundance and diversity during the wet season due to improved access to available nutrients (Hu et al., 2001) and the stimulation of bacterial migration (Taketani et al., 2017). In contrast to the bacterial community, the fungal community diversity and species richness remained constant under increasing precipitation (Supplementary Tables 7, 8). This confirmed the hypothesis of Barnard et al. (2013) that soil fungal communities were unaltered by multiple dry–wet periods, which exhibited a clear resistance to variations in the soil moisture content. These distinct fungal and bacterial responses explained the disparities between bacteria and fungi in ambient ecological environments (Boer et al., 2005), which showed that karst ecosystems contain various types of water-related niches.

## Conclusion

The karst regions of Southwestern China are undergoing climate-induced changes, including increased precipitation and higher soil moisture, which are likely to intensify in the near future. Our results clearly revealed the differential effects of water addition on bacterial and fungal communities during the dry and wet seasons. First, water addition could result in the significant alterations in elemental concentrations and the pH of soils in karst areas across both seasons. Second, during the dry season, water addition did not cause any significant changes in the compositions of neither bacterial nor fungal communities. However, during the wet season, water addition caused a significant variation in the relative abundance of some bacterial phyla but not for fungal phyla. This overstates differential responses from bacteria and fungi to increasing rainfall. Third, during the wet season, the structure and diversity of bacterial communities were positively associated with soil K and Na, which was increased with water addition. These findings suggested that increasing precipitation might cause multifaceted effects on microbial populations and associated biogeochemistry in karst areas, which should be taken into consideration toward coping with climate change in this region.

Changes in rainfall regimes that are predicted to have a significant impact on degraded karst ecosystems may alter the living history of soil organisms, which in turn may have an effect on microbial population and its characteristics, with potentially large-scale impacts on nutrient cycling and carbon budgets. Such information is extremely important for the restoration of the degraded karst ecosystems, as the rich bacterial diversity during the wet season can greatly support tree seedlings and their growth. Hence, there is a great need for studies taking a more whole systems approach to quantifying effects of increased precipitation in degraded karst ecosystems.

## Data Availability Statement

The datasets presented in this study can be found in online repositories. The names of the repository/repositories and accession number(s) can be found in the article/ Supplementary Material.

## Author Contributions

CL and MU designed the study. MU, NS, and CL collected field samples and data. MU, CL, and NH analyzed the data. MU wrote the manuscript. NH, MA, CL, BD, and HD revised the manuscript. All authors contributed to the article and approved the submitted version.

## Conflict of Interest

The authors declare that the research was conducted in the absence of any commercial or financial relationships that could be construed as a potential conflict of interest.

## Footnotes

1 http://data.cma.cn/en/?r=data/weatherBk

2 www.ncbi.nlm.nih.gov
