# Supplementary material for: Bacterial Communities Are More Sensitive to Water Addition Than Fungal Communities Due to Higher Soil K and Na in a Degraded Karst Ecosystem of Southwestern China
Source: Front Microbiol. 2020 Nov 9;11:562546. doi: 10.3389/fmicb.2020.562546 (PMC7680866; doi:10.3389/fmicb.2020.562546)
Supplement: Supplementary file 1 [file Data_Sheet_1.docx]

**Bacterial communities are more sensitive to water addition than fungal communities due to higher soil K and Na in a degraded karst ecosystem of Southwestern China**

Muhammad Umair^1^, Ningxiao Sun^1,2,3^, Hongmei Du^4^, Nan Hui^1^, Muhammad Altaf^5^, Baoming Du^1,2,3^, Shan Yin^1,2,3^, Chunjiang Liu^1,2,3*^

^1^School of Agriculture and Biology, Shanghai Jiao Tong University, Shanghai, 200240, China

^2^Shanghai Urban Forest Research Station, State Forestry and Grassland Administration, Shanghai, China

^3^Key Laboratory of Urban Agriculture (South), Ministry of Agriculture, Shanghai, China

^4^School of Design, Shanghai Jiao Tong University, Shanghai, 200240, China

^5^Department of Zoology, Women University of Azad Jammu and Kashmir, Bagh, 12150, Pakistan

**Correspondence emails:**

[chjliu@sjtu.edu.cn](mailto:chjliu@sjtu.edu.cn；hmdu@sjtu.edu.cn)

**
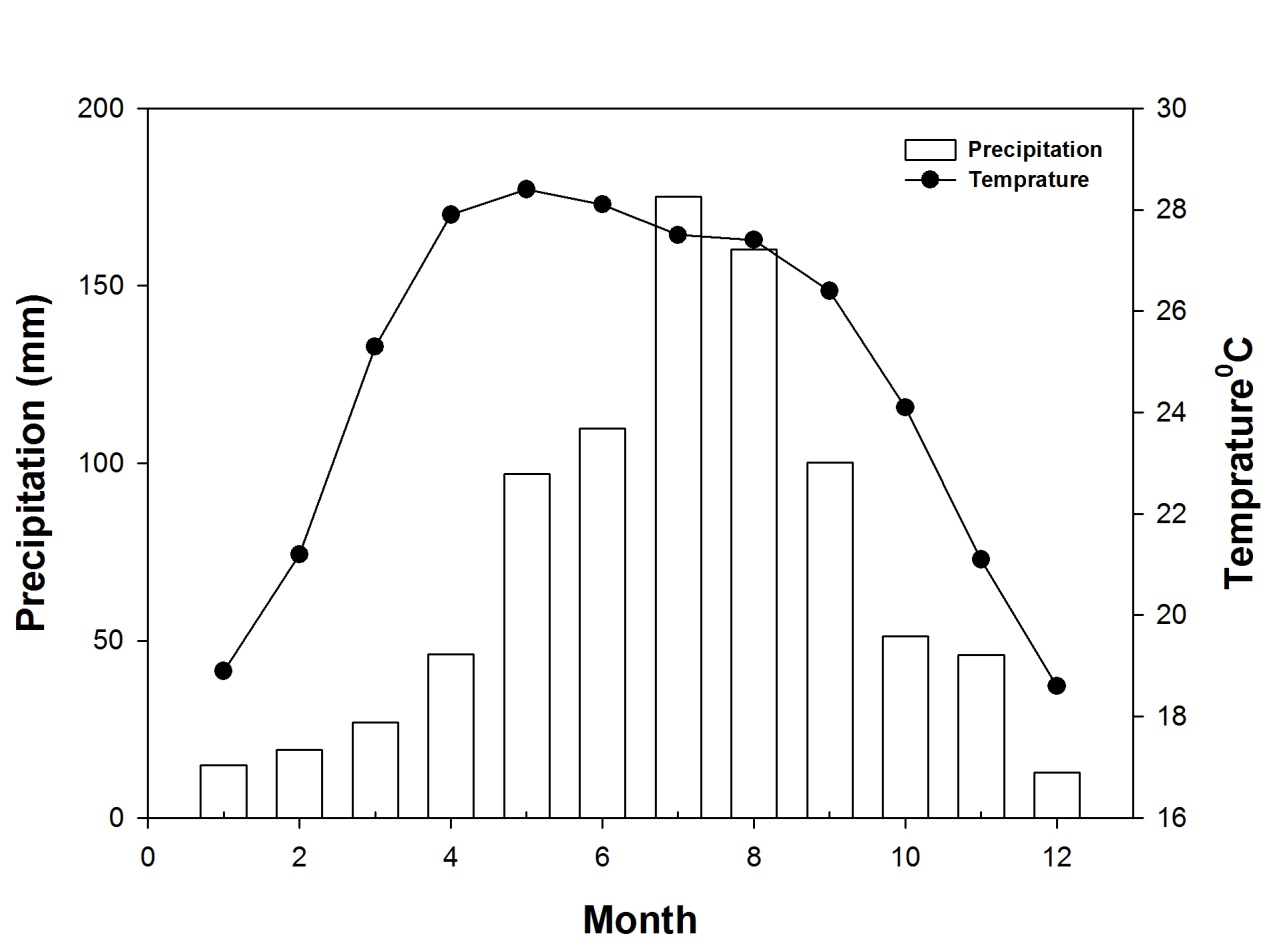
**

**Supplementary Figure 1.** The distribution of average monthly precipitation and average monthly temperature from 2010 to 2017.

**
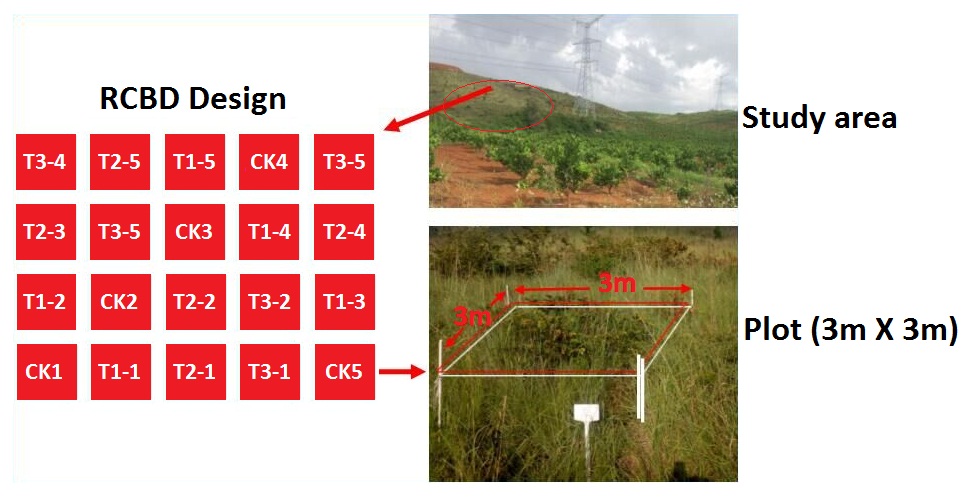
**

**Supplementary Figure 2.** Plotting the study area for water treatments, 3m × 3m quadrates with 1 control, 3 treatments in a karst area of southwestern China.

**Supplementary Figure 3.** Daily variation in precipitation (mm) during the study period.

**Supplementary Figure 4.** Daily variation in air temperature (^0^C) during the study period.

**
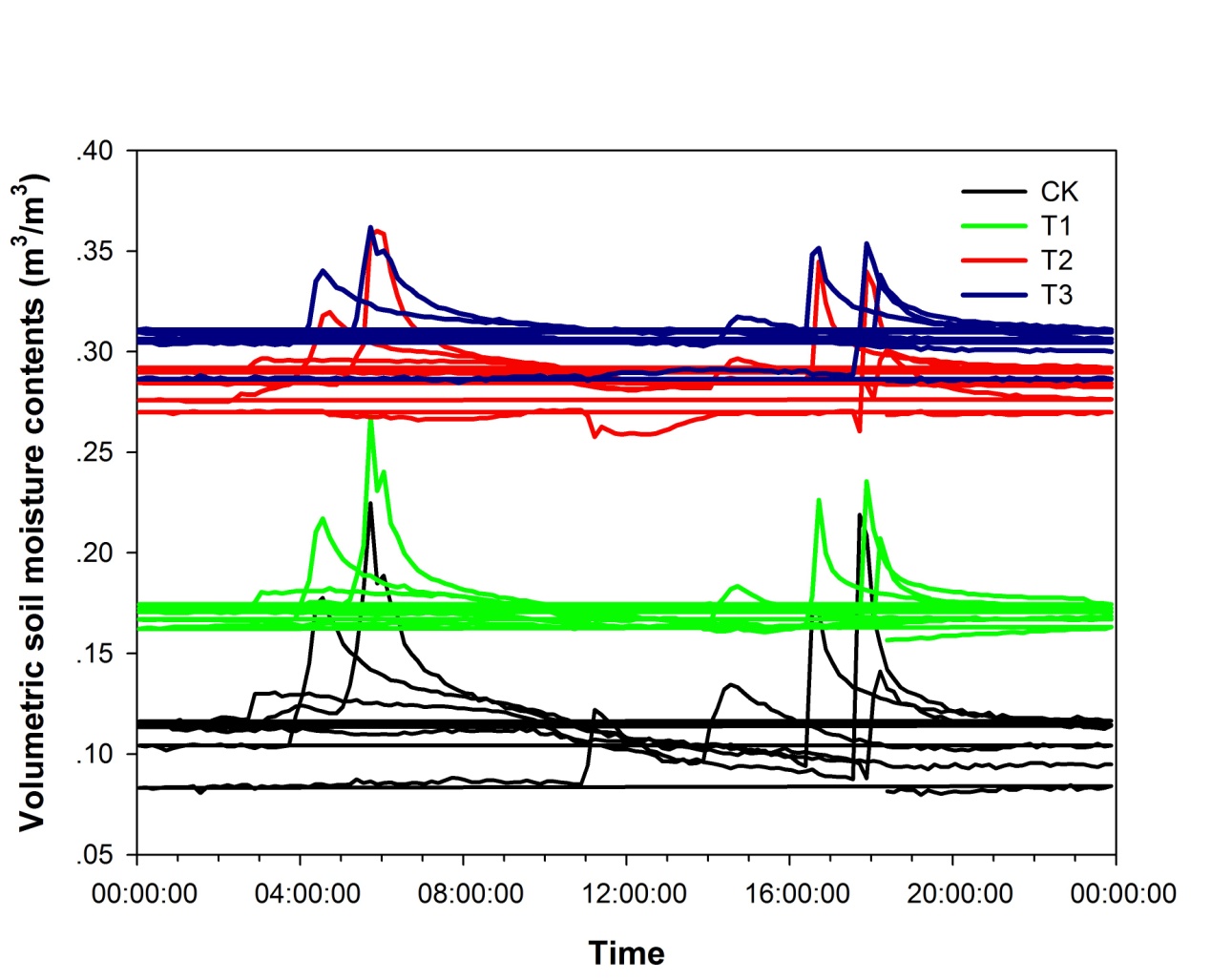
**

**Supplementary Figure 5.** Daily variations in the volumetric soil moisture contents (m^3^/m^3^) across four watering treatments during the study period.

**
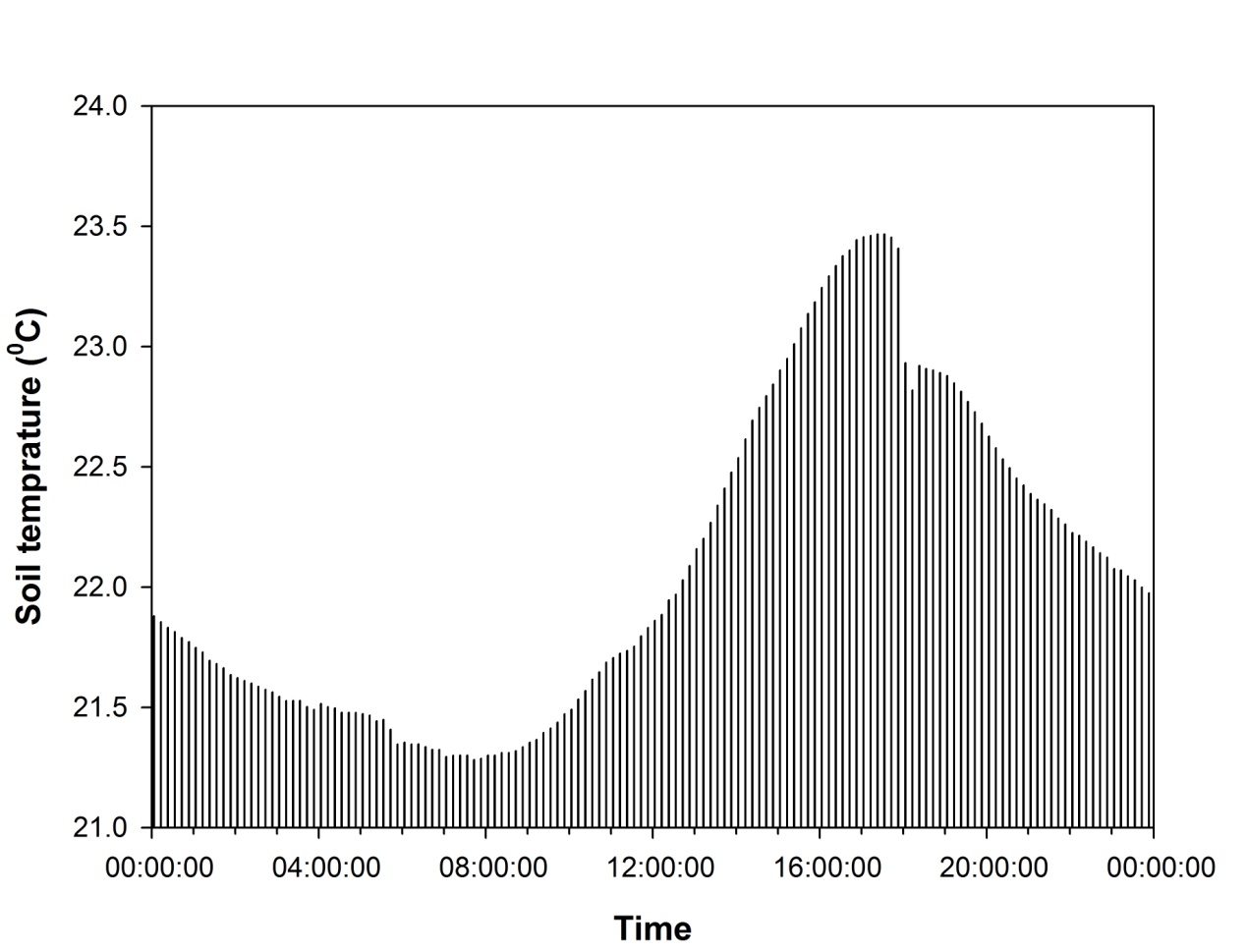
**

**Supplementary Figure 6.** Daily variations of average soil temperatures during the study period.

**Supplementary Figure 7.** Daily variations in average air temperature (Air Temp.) and in average soil temperature (soil Temp) at 00:00 -24:00.


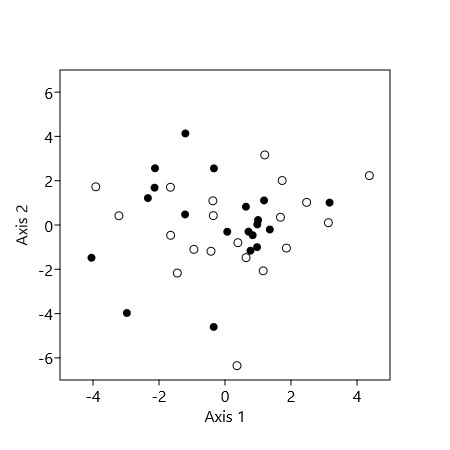


**Supplementary Figure 8** Principal component analysis demonstrated the soil elemental variables of two seasons under 0% (CK), +20% (T1), +40% (T2) and +60% (T3) watering treatments. The elemental variables of soils were mixed between seasons (*p* <0.7).
