# Supplementary material for: Bacterial Communities Are More Sensitive to Water Addition Than Fungal Communities Due to Higher Soil K and Na in a Degraded Karst Ecosystem of Southwestern China
Source: Front Microbiol. 2020 Nov 9;11:562546. doi: 10.3389/fmicb.2020.562546 (PMC7680866; doi:10.3389/fmicb.2020.562546)
Supplement: Supplementary file 2 [file Data_Sheet_2.docx]

**Bacterial communities are more sensitive to water addition than fungal communities due to higher soil K and Na in a degraded karst ecosystem of Southwestern China**

Muhammad Umair^1^, Ningxiao Sun^1,2,3^, Hongmei Du^4^, Nan Hui^1^, Muhammad Altaf^5^, Baoming Du^1,2,3^, Shan Yin^1,2,3^, Chunjiang Liu^1,2,3*^

^1^School of Agriculture and Biology, Shanghai Jiao Tong University, Shanghai, 200240, China

^2^Shanghai Urban Forest Research Station, State Forestry and Grassland Administration, Shanghai, China

^3^Key Laboratory of Urban Agriculture (South), Ministry of Agriculture, Shanghai, China

^4^School of Design, Shanghai Jiao Tong University, Shanghai, 200240, China

^5^Department of Zoology, Women University of Azad Jammu and Kashmir, Bagh, 12150, Pakistan

**Correspondence emails:**

[chjliu@sjtu.edu.cn](mailto:chjliu@sjtu.edu.cn)

**Supplementary Table 1.** Loading of elemental and stoichiometric variables of twenty soil samples in PCA analysis

| **Soil variables** | **Axis 1** | **Axis 2** | **Axis 3** |
| --- | --- | --- | --- |
| pH | -0.13 | -0.35 | 0.24 |
| SWC (%) | 0.59 | -0.05 | 0.78 |
| N | 0.42 | 0.84 | 0.16 |
| C | -0.51 | 0.78 | 0.20 |
| H | 0.30 | 0.00 | 0.81 |
| S | -0.40 | 0.55 | -0.42 |
| Fe | 0.72 | -0.60 | -0.20 |
| P | 0.44 | 0.64 | -0.53 |
| K | 0.68 | 0.13 | -0.64 |
| Ca | -0.17 | 0.81 | 0.18 |
| Mg | 0.93 | 0.17 | -0.21 |
| Mn | 0.50 | 0.73 | -0.06 |
| Zn | -0.55 | 0.72 | 0.24 |
| Cu | 0.65 | 0.32 | 0.10 |
| Na | 0.91 | 0.11 | 0.07 |
| Al | 0.65 | -0.61 | 0.12 |
| Si | 0.72 | 0.60 | 0.27 |
| % variance | 34.39 | 30.011 | 14.881 |
| Eigen value | 5.84625 | 5.10182 | 2.5297 |
| *p* season | 0.14 | 0.77 | **<0.001** |
| *p* water | **<0.001** | 0.05 | 0.16 |
| *p* water $\times$ interaction | 0.82 | 0.26 | 0.04 |

**Supplementary Table 2.** Means ± SE of microbial phyla in both dry and wet season under the 0% (CK), +20% (T1), +40% (T2) and +60% (T3) watering treatments. The values in bold indicate statistical significance at *p* < 0.05 in one-way ANOVA.

| **Microbial phyla** | **Dry season** | | | | **Wet season** | | | | F and *p* values |
| --- | --- | --- | --- | --- | --- | --- | --- | --- | --- |
|  | CK | T1 | T2 | T3 | CK | T1 | T2 | T3 |  |
| Actinobacteria | 0.549±0.046a | 0.528±0.024a | 0.512±0.026a | 0.512±0.023a | 0.367±0.013b | 0.427±0.014ab | 0.409±0.027ab | 0.444±0.023a | **37.51 (<0.01)** |
| Proteobacteria | 0.196±0.024a | 0.221±0.004a | 0.212±0.009a | 0.228±0.015a | 0.213±0.016a | 0.215±0.029a | 0.272±0.005a | 0.267±0.015a | **4.43 (0.04)** |
| Acidobacteria | 0.093±0.016a | 0.095±0.010a | 0.103±0.009a | 0.099±0.009a | 0.160±0.007a | 0.158±0.015a | 0.124±0.018b | 0.112±0.010b | **19.71 (<0.01)** |
| Chloroflexi | 0.084±0.005a | 0.081±0.008a | 0.086±0.006a | 0.068±0.003a | 0.079±0.011a | 0.102±0.014a | 0.078±0.006a | 0.079±0.003a | 0.57 (0.45) |
| Planctomycetes | 0.017±0.002a | 0.018±0.002a | 0.018±0.003a | 0.019±0.004a | 0.017±0.003a | 0.016±0.002a | 0.014±0.002ab | 0.008±0.002b | **5.45 (0.02)** |
| Nitrospirae | 0.007±0.001a | 0.007±0.001a | 0.007±0.001a | 0.007±0.002a | 0.011±0.002a | 0.012±0.002a | 0.009±0.002a | 0.008±0.001a | **8.98 (<0.01)** |
| Bacteroidetes | 0.005±0.001a | 0.004±0.001a | 0.005±0.001a | 0.005±0.001a | 0.009±0.001ab | 0.008±0.002b | 0.013±0.001a | 0.013±0.003ab | **27.40 (<0.01)** |
| Firmicutes | 0.002±0.000a | 0.005±0.002a | 0.007±0.005a | 0.004±0.001a | 0.007±0.002a | 0.009±0.002a | 0.010±0.001a | 0.008±0.003a | **7.92 (0.01)** |
| Verrucomicrobia | 0.004±0.002a | 0.004±0.000a | 0.005±0.001a | 0.004±0.001a | 0.017±0.008a | 0.006±0.001b | 0.011±0.004ab | 0.003±0.001b | **3.99 (0.049)** |
| Ascomycota | 0.827±0.037a | 0.842±0.037a | 0.885±0.040a | 0.800±0.088a | 0.711±0.642a | 0.688±0.790a | 0.072±0.121a | 0.100±0.065a | **6.54 (0.01)** |
| Basidiomycota | 0.101±0.037a | 0.077±0.024a | 0.072±0.041a | 0.090±0.048a | 0.210±0.305a | 0.178±0.104a | 0.055±0.117a | 0.065±0.027a | **7.53 (<0.01)** |
| Mortierellomycota | 0.0008±0.0003a | 0.0095±0.0066a | 0.0007±0.0005a | 0.0002±0.0001a | 0.023±0.008a | 0.013±0.007a | 0.013±0.006a | 0.010±0.005a | **4.48 (0.04)** |
| Chytridiomycota | 0.0002±0.0001a | 0.0019±0.0011a | 0.0001±0.0001a | 0.0006±0.0005a | 0.006±0.005a | 0.003±0.002a | 0.004±0.004a | 0.001±0.001a | **5.12 (0.03)** |
| Glomeromycota | 0.0006±0.0004a | 0.0009±0.0005a | 0.0000±0.0000a | 0.0001±0.0001a | 0.003±0.001a | 0.002±0.001a | 0.002±0.001a | 0.001±0.000a | 3.92 (0.05) |

†Values with different letters indicated significant difference between treatments detected by Duncan's Multiple Range Test (*P* < 0.05).

**Supplementary Table 3.** Indicator species analysis showing bacterial genera significantly associated to watering treatment groups across seasons in a karst area, southwestern China. Indicator values (%) are given in the table. Asterisk showed the significant *p* value.

| **Season** | **Bacterial Phyla** | **Bactarial genera** | CK1 | T1 | T2 | T3 | CK+T1 | T1+T2 | T2+T3 | CK+T1+T2 | CK+T2+T3 | T1+T2+T3 |
| --- | --- | --- | --- | --- | --- | --- | --- | --- | --- | --- | --- | --- |
| **Dry season** | Verrucomicrobia | *OPB35* bacterium |  |  | 91.0 ** |  |  |  |  |  |  |  |
|  | Proteobacteria | *Chondromyces* |  |  |  |  | 80.1 * |  |  |  |  |  |
|  | Actinobacteria | *Frankia* |  |  |  |  |  |  |  | 97.6 ** |  |  |
|  |  |  |  |  |  |  |  |  |  |  |  |  |
| **Wet season** | Firmicutes | *NK4A136_*group |  |  | 78.8 * |  |  |  |  |  |  |  |
|  | Proteobacteria | *Erythrobacter* |  |  |  | 20.0 * |  |  |  |  |  |  |
|  | Proteobacteria | *Methylosinus* |  |  |  | 28.0 * |  |  |  |  |  |  |
|  | Proteobacteria | *Cystobacter* |  |  |  | 43.0 * |  |  |  |  |  |  |
|  | Actinobacteria | *Planosporangium* |  |  |  |  | 15.0 * |  |  |  |  |  |
|  | Bacteroidetes | *Spirosoma* |  |  |  |  |  |  | 37.0 * |  |  |  |
|  | Acidobacteria | *Elev-16S-573* |  |  |  |  |  |  |  | 36.0 * |  |  |
|  | Proteobacteria | *Pelomonas* |  |  |  |  |  |  |  | 32.0 * |  |  |
|  | Proteobacteria | *Dokdonella* |  |  |  |  |  |  |  |  | 22.0 * |  |
|  | Proteobacteria | *Novosphingobium* |  |  |  |  |  |  |  |  |  | 88.4 * |
|  | Actinobacteria | *Quadrisphaera* |  |  |  |  |  |  |  |  |  | 85.6 * |
|  | Actinobacteria | *Sphaerisporangium* |  |  |  |  |  |  |  |  |  | 84.4 * |

*** indicates significance (p *<*0.05); **** indicates high significance (p *<*0.01).

**Supplementary Table 4.** Indicator species analysis showing fungal genera significantly associated to watering treatment groups across seasons in a karst area, Southwestern China. Indicator values (%) are given in the table. Asterisk showed the significant *p* value.

| **Season** | **Bacterial Phyla** | **Bacterial genera** | **CK1** | **T1** | **T2** | **T3** | **CK+T1** | **CK+T2** | **T1+T2** | **T1+T3** | **T2+T3** | **CK+T1+T2** | **CK+T1+T3** | **T1+T2+T3** |
| --- | --- | --- | --- | --- | --- | --- | --- | --- | --- | --- | --- | --- | --- | --- |
| **Dry Season** | Basidiomycota | *Tricholoma* | 93.7 ** |  |  |  |  |  |  |  |  |  |  |  |
|  | Basidiomycota | *Neonothopanus* | 76.0 * |  |  |  |  |  |  |  |  |  |  |  |
|  | Chytridiomycota | *Rhizophlyctis* |  | 78.8 * |  |  |  |  |  |  |  |  |  |  |
|  | Ascomycota | *Viridispora* |  | 77.5 * |  |  |  |  |  |  |  |  |  |  |
|  | Ascomycota | *Aciculosporium* |  |  |  |  | 99.1 *** | |  |  |  |  |  |  |
|  | Ascomycota | *Coniothyrium* |  |  |  |  |  |  | 91.3 * |  |  |  |  |  |
|  | Ascomycota | *Archaeorhizomyces* |  |  |  |  |  |  |  |  |  | 98.8 ** |  |  |
|  | Ascomycota | *Ophiocordyceps* |  |  |  |  |  |  |  |  |  | 21.0 * |  |  |
|  | Basidiomycota | *Clavaria* |  |  |  |  |  |  |  |  |  | 14.0 * |  |  |
|  | Ascomycota | *Monilinia* |  |  |  |  |  |  |  |  |  | 43.0 * |  |  |
|  | Ascomycota | *Aureobasidium* |  |  |  |  |  |  |  |  |  |  | 95.2 ** |  |
|  | Ascomycota | *Phoma* |  |  |  |  |  |  |  |  |  |  | 95.0 * |  |
|  | Ascomycota | *Thaxteriellopsis* |  |  |  |  |  |  |  |  |  |  |  | 89.4 * |
|  |  |  |  |  |  |  |  |  |  |  |  |  |  |  |
| **Wet Season** | Ascomycota | *Chaetomium* |  | 90.0 * |  |  |  |  |  |  |  |  |  |  |
|  | Ascomycota | *Sclerostagonospora* |  | 43.0 * |  |  |  |  |  |  |  |  |  |  |
|  | Ascomycota | *Ampelomyces* |  |  | 40.0 * |  |  |  |  |  |  |  |  |  |
|  | Ascomycota | *Sporormiella* |  |  |  | 73.5 * |  |  |  |  |  |  |  |  |
|  | Ascomycota | *Physalospora* |  |  |  |  |  | 83.3 * |  |  |  |  |  |  |
|  | Ascomycota | *Ilyonectria* |  |  |  |  |  |  |  | 83.9 * |  |  |  |  |
|  | Ascomycota | *Tiarosporella* |  |  |  |  |  |  |  |  | 89.4 * |  |  |  |
|  | Chlorophyta | *Trebouxia* |  |  |  |  |  |  |  |  |  |  |  | 98.8 ** |
|  | Ascomycota | *Petrakia* |  |  |  |  |  |  |  |  |  |  |  | 85.6 * |

*** indicates significance (p *<*0.05); **** indicates high significance (p *<*0.01); ***** indicates very high significance (p *<*0.001).

**Supplementary Table 5**. Loading of microbial community structure and soil elemental variables in CCA analysis.

| **Microbial communities and soil chemical variables** | **Axis 1** | **Axis 2** |
| --- | --- | --- |
| Actinobacteria | -0.14 | -0.81 |
| Proteobacteria | 0.02 | -0.48 |
| Acidobacteria | 0.37 | 1.49 |
| Chloroflexi | 0.05 | 0.09 |
| Planctomycetes | 0.39 | 3.21 |
| Nitrospirae | 0.43 | 1.82 |
| Bacteroidetes | -0.22 | -0.20 |
| Firmicutes | 0.05 | 0.10 |
| Verrucomicrobia | 1.11 | 10.41 |
| Ascomycota | -0.77 | 0.20 |
| Basidiomycota | 2.63 | -0.28 |
| pH | -0.02 | -0.19 |
| SWC % | -0.28 | -0.23 |
| N | -0.25 | -0.31 |
| C | 0.13 | -0.02 |
| H | -0.16 | -0.10 |
| S | 0.19 | 0.03 |
| Fe | -0.23 | -0.10 |
| P | -0.26 | -0.25 |
| K | -0.44 | -0.48 |
| Ca | -0.37 | -0.23 |
| Mg | -0.37 | -0.39 |
| Mn | -0.21 | -0.17 |
| Zn | 0.06 | 0.27 |
| Cu | -0.28 | 0.04 |
| Na | -0.49 | -0.49 |
| Al | -0.27 | 0.03 |
| Si | -0.32 | -0.36 |
| Eigen value | 0.08 | 0.01 |
| % variance | 79.86 | 7.94 |
| *p* value | 0.36 | **0.02** |

**Supplementary Table 6.** Pearson correlation coefficient between soil variables and microbial phyla of both seasons. The significant ‘r’ values are bold (*p* < 0.05).

| **Soil microbial phyla** | **Dry Season** | | | | | | | | | | | | | | | | |
| --- | --- | --- | --- | --- | --- | --- | --- | --- | --- | --- | --- | --- | --- | --- | --- | --- | --- |
|  | **pH** | **SWC** | **N** | **C** | **H** | **S** | **Fe** | **P** | **K** | **Ca** | **Mg** | **Mn** | **Zn** | **Cu** | **Na** | **Al** | **Si** |
| **Actinobacteria** | -0.10 | -0.24 | -0.19 | -0.19 | -0.03 | -0.19 | -0.18 | -0.20 | 0.02 | -0.18 | -0.27 | -0.13 | 0.40 | 0.03 | -0.30 | -0.22 | 0.21 |
| **Proteobacteria** | -0.19 | 0.25 | -0.05 | -0.13 | -0.19 | -0.08 | 0.35 | 0.03 | 0.14 | -0.15 | 0.33 | 0.25 | -0.35 | 0.05 | 0.20 | 0.35 | 0.29 |
| **Acidobacteria** | 0.08 | 0.19 | 0.29 | 0.30 | 0.05 | 0.27 | 0.16 | 0.28 | -0.09 | 0.32 | 0.26 | 0.16 | -0.32 | -0.05 | 0.31 | 0.15 | -0.34 |
| **Chloroflexi** | **0.51** | -0.28 | 0.32 | 0.37 | 0.01 | 0.33 | -0.25 | 0.05 | 0.00 | 0.41 | 0.00 | 0.05 | 0.12 | 0.16 | -0.09 | -0.25 | **-0.63** |
| **Planctomycetes** | 0.19 | 0.25 | 0.22 | 0.28 | 0.27 | 0.26 | 0.03 | 0.16 | -0.09 | 0.31 | 0.10 | -0.10 | -0.32 | -0.02 | 0.23 | 0.09 | -0.27 |
| **Nitrospirae** | 0.01 | 0.24 | 0.14 | 0.24 | 0.23 | 0.27 | -0.13 | 0.10 | -0.09 | 0.29 | -0.11 | -0.25 | -0.32 | -0.18 | 0.15 | 0.02 | -0.16 |
| **Bacteroidetes** | 0.07 | 0.29 | 0.27 | 0.31 | 0.29 | 0.21 | 0.17 | 0.40 | 0.04 | 0.28 | 0.35 | 0.33 | -0.33 | 0.03 | 0.27 | 0.16 | -0.17 |
| **Firmicutes** | 0.09 | 0.17 | 0.13 | 0.09 | 0.13 | 0.19 | -0.05 | 0.07 | 0.32 | -0.10 | 0.10 | 0.04 | -0.30 | 0.20 | 0.00 | 0.18 | -0.02 |
| **Verrucomicrobia** | -0.02 | 0.23 | -0.04 | 0.10 | 0.14 | 0.05 | 0.06 | 0.13 | -0.20 | 0.03 | -0.05 | -0.29 | -0.43 | -0.33 | 0.19 | 0.11 | -0.18 |
| **Ascomycota** | -0.16 | 0.00 | -0.01 | 0.05 | 0.20 | 0.05 | 0.08 | 0.02 | -0.21 | 0.04 | -0.15 | -0.43 | -0.40 | -0.14 | 0.05 | 0.25 | -0.11 |
| **Basidiomycota** | 0.18 | 0.04 | 0.19 | 0.15 | 0.09 | 0.12 | -0.02 | 0.17 | 0.13 | 0.16 | 0.25 | **0.46** | 0.27 | 0.12 | 0.14 | -0.20 | -0.14 |
|  | **Wet season** | | | | | | | | | | | | | | | | |
| **Actinobacteria** | 0.30 | 0.28 | **0.49** | 0.05 | 0.15 | 0.03 | 0.01 | 0.22 | **0.84** | **0.60** | 0.39 | 0.17 | -0.07 | 0.20 | **0.54** | -0.13 | 0.43 |
| **Proteobacteria** | -0.08 | **0.47** | 0.15 | 0.05 | 0.27 | 0.02 | 0.29 | 0.07 | 0.26 | -0.15 | 0.26 | 0.15 | 0.07 | 0.27 | **0.47** | 0.29 | 0.03 |
| **Acidobacteria** | -0.05 | -0.43 | **-0.60** | -0.22 | -0.42 | -0.12 | -0.33 | **-0.48** | **-0.60** | -0.34 | **-0.66** | **-0.57** | -0.14 | -0.35 | **-0.60** | -0.15 | -0.26 |
| **Chloroflexi** | 0.26 | -0.27 | -0.38 | -0.20 | -0.28 | -0.09 | -0.31 | -0.31 | 0.02 | -0.08 | -0.34 | -0.44 | -0.12 | -0.12 | -0.24 | -0.20 | 0.00 |
| **Planctomycetes** | 0.11 | -0.28 | **-0.59** | -0.12 | -0.19 | 0.11 | -0.26 | **-0.52** | -0.39 | -0.30 | **-0.57** | **-0.48** | 0.00 | 0.01 | **-0.49** | 0.02 | **-0.64** |
| **Nitrospirae** | 0.17 | -0.15 | **-0.58** | -0.25 | -0.27 | 0.03 | -0.35 | **-0.52** | -0.22 | -0.16 | **-0.51** | **-0.64** | -0.23 | -0.03 | -0.36 | -0.02 | -0.29 |
| **Bacteroidetes** | 0.13 | 0.43 | 0.07 | -0.09 | 0.21 | -0.07 | 0.42 | 0.17 | 0.15 | -0.22 | **0.44** | 0.34 | 0.42 | **0.59** | **0.53** | **0.48** | -0.08 |
| **Firmicutes** | 0.16 | 0.02 | -0.29 | -0.20 | -0.18 | -0.34 | -0.03 | -0.25 | 0.06 | -0.25 | -0.34 | -0.39 | -0.14 | -0.25 | -0.12 | -0.07 | **0.45** |
| **Verrucomicrobia** | -0.21 | -0.11 | -0.02 | 0.27 | 0.10 | 0.27 | -0.07 | -0.13 | -0.41 | -0.16 | -0.22 | 0.06 | **0.50** | 0.13 | -0.41 | -0.09 | -0.37 |
| **Ascomycota** | 0.01 | 0.18 | 0.21 | -0.17 | 0.09 | -0.25 | 0.23 | 0.22 | **0.46** | 0.26 | 0.28 | 0.25 | 0.00 | 0.30 | 0.38 | 0.27 | 0.28 |
| **Basidiomycota** | -0.02 | -0.30 | -0.21 | 0.14 | -0.16 | 0.19 | -0.22 | -0.22 | **-0.52** | -0.36 | -0.35 | -0.14 | 0.07 | -0.27 | **-0.48** | -0.27 | -0.31 |

**Supplementary Table 7.** Means ± SE of species richness and bacterial diversity indices of two seasons under the 0% (CK), +20% (T1), +40% (T2) and +60% (T3) watering treatments.

| **Treatment** | **Dry season** | | | | |
| --- | --- | --- | --- | --- | --- |
|  | **Chao** | **OTUs** | **Shannon (H)** | **Simpson (1/D)** | **Evenness** |
| CK | 5314±222a | 4021±242a | 6.72±0.09a | 162±16a | 0.04±0.003a |
| T1 | 5398±140a | 4081±159a | 6.75±0.05a | 144±10a | 0.035±0.002a |
| T2 | 5477±158a | 4111±171a | 6.74±0.06a | 142±20a | 0.034±0.004a |
| T3 | 4922±169a | 3665±215a | 6.7±0.06a | 150±20a | 0.041±0.005a |
|  | **Wet season** | | | | |
| CK | 6504±283b | 4689±220c | 7.16±0.42a | 471±133a | 0.098±0.026a |
| T1 | 6693±344ab | 4877±292bc | 7.54±0.16a | 521±105a | 0.103±0.019a |
| T2 | 7191±207ab | 5515±275ab | 7.71±0.03a | 633±86a | 0.118±0.02a |
| T3 | 7415±93a | 6108±113a | 7.76±0.02a | 622±42a | 0.102±0.008a |

†Values with different letters indicated significant difference between treatments detected by Duncan's Multiple Range Test (*P* < 0.05).

**Supplementary Table 8.** Means ± SE of species richness and fungal diversity indices of two seasons under the 0% (CK), +20% (T1), +40% (T2) and +60% (T3) watering treatments.

| **Treatment** | **Dry season** | | | | |
| --- | --- | --- | --- | --- | --- |
|  | **chao** | **OTUs** | **Shannon (H)** | **Simpson (1/D)** | **Evenness** |
| CK | 1808±97a | 4996±316ab | 4.43±0.27ab | 23.26±5.93ab | 0.018±0.004ab |
| T1 | 1804±124a | 5285±216a | 4.93±0.19a | 44.97±12.47a | 0.036±0.012a |
| T2 | 1776±121a | 4238±361b | 3.9±0.46b | 16.97±5.48b | 0.013±0.004b |
| T3 | 1728±178a | 4511±567ab | 4.46±0.33ab | 30.05±7.51ab | 0.022±0.004ab |
|  | **Wet season** | | | | |
| CK | 1147±236a | 4189±141a | 4.55±0.18a | 38.17±8.92a | 0.061±0.024a |
| T1 | 1546±256a | 4369±172a | 3.78±0.57a | 22.03±11.46a | 0.017±0.007a |
| T2 | 1237±220a | 4060±74a | 4.42±0.16a | 26.88±4.24a | 0.033±0.008a |
| T3 | 1359±134a | 4504±84a | 4.4±0.24a | 27.37±6.26a | 0.03±0.008a |

†Values with different letters indicated significant difference between treatments detected by Duncan's Multiple Range Test (*P* < 0.05).
